# Proteomic mapping reveals dysregulated angiogenesis in the cerebral arteries of rats with early-onset hypertension

**DOI:** 10.1016/j.jbc.2023.105221

**Published:** 2023-09-01

**Authors:** Joakim A. Bastrup, Thomas A. Jepps

**Affiliations:** Department of Biomedical Sciences, University of Copenhagen, Copenhagen, Denmark

**Keywords:** hypertension, angiogenesis, cerebrovascular disease, cerebral arteries, DIA-MS

## Abstract

Hypertension is associated with the presence of vascular abnormalities, including remodeling and rarefaction. These processes play an important role in cerebrovascular disease development; however, the mechanistic changes leading to these diseases are not well characterized. Using data-independent acquisition–based mass spectrometry analysis, here we determined the protein changes in cerebral arteries in pre- and early-onset hypertension from the spontaneously hypertensive rat (SHR), a model that resembles essential hypertension in humans. Our analysis identified 125 proteins with expression levels that were significantly upregulated or downregulated in 12-week-old spontaneously hypertensive rats compared to normotensive Wistar Kyoto rats. Using an angiogenesis enrichment analysis, we further identified a critical imbalance in angiogenic proteins that promoted an anti-angiogenic profile in cerebral arteries at early onset of hypertension. In a comparison to previously published data, we demonstrate that this angiogenic imbalance is not present in mesenteric and renal arteries from age-matched SHRs. Finally, we identified two proteins (Fbln5 and Cdh13), whose expression levels were critically altered in cerebral arteries compared to the other arterial beds. The observation of an angiogenic imbalance in cerebral arteries from the SHR reveals critical protein changes in the cerebrovasculature at the early onset of hypertension and provides novel insights into the early pathology of cerebrovascular disease.

Many factors are involved in the development of hypertension, which is primarily associated with an increase in the total peripheral resistance, advocating for the presence of vascular abnormalities. Such abnormalities include structural changes to the arterial wall (remodeling and rarefication) (1), altered excitation-contraction coupling, and changes in the neurogenic and humoral signaling (2). In hypertension, a general narrowing of the large resistance arteries through to the precapillary microcirculation is associated with eutrophic and hypertrophic remodeling of the arterial wall in most vascular beds (3, 4, 5). We, and others, have demonstrated that arterial remodeling occurs in certain systemic arteries soon after the onset of hypertension (12-weeks-old) in the spontaneously hypertensive rat (SHR) (6), a model that is considered to resemble the main features of essential hypertension in humans without confounding lifestyle and environmental factors (7). Although the remodeling processes are key features of hypertension, the mechanistic pathways involved are not understood fully.

In-depth quantitative proteomics can achieve unique insight into complex biological mechanisms and pathologies (8, 9). We have previously optimized a label-free proteomic workflow for analyzing resistance arteries in the SHR, resulting in the identification of more than 4700 unique proteins (7). Using gene-overrepresentation analysis, these proteins provided novel mechanistic insight into different biological pathways that were altered during the initiation of systemic arterial remodeling, such as changes in the extracellular matrix (ECM) (7).

Cerebrovascular diseases such as stroke, cognitive decline, and vascular dementia are highly associated with hypertension (10, 11, 12). There is evidence that the media-to-lumen ratios of cerebral arteries from the SHR do not display the same level of remodeling as systemic vessels (13); however, the link between hypertension and cerebrovascular diseases suggests there is a profound effect of hypertension on cerebral arteries (14). Thus, the aim of this study was to uncover protein changes and mechanistic pathways that are altered in cerebral arteries from hypertensive rats. By determining the hypertension-induced dysregulated pathways in cerebral arteries, we can better understand how hypertension increases the risk of cerebrovascular diseases. Using label-free in-depth proteomic profiling, we reveal that the cerebral arteries from SHRs with early-onset hypertension have several dysregulated proteins associated with angiogenesis, which is not seen in mesenteric or renal arteries of age-matched SHRs.

## Results

### Cerebral arteries in early-onset hypertensive rats display increased wall remodeling

**Figure 1:**
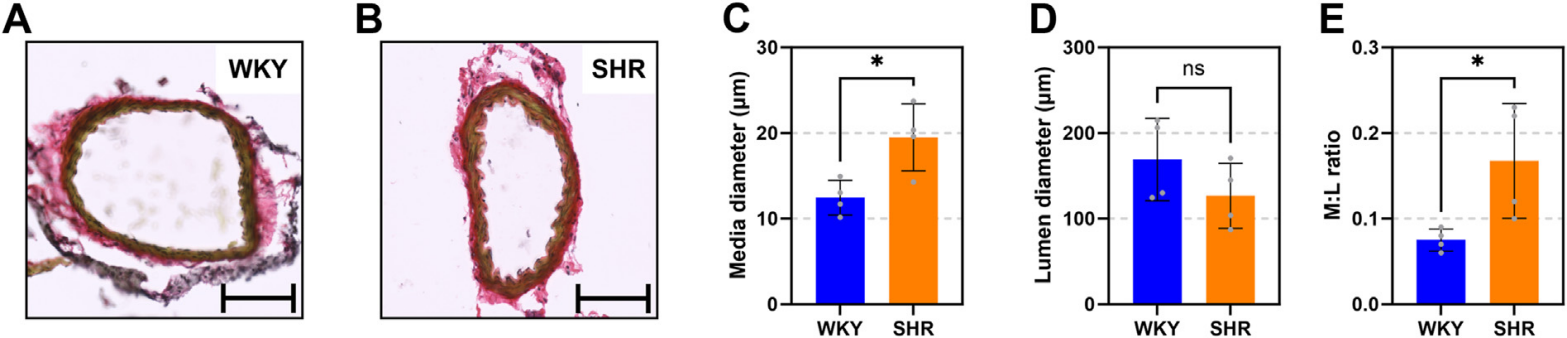
**Vascular remodeling in cerebral arteries.***A* and *B*, exemplary Sirius red stained basilar arteries from a subgroup of 12-week-old WKY (*A*) or SHR (*B*) rats (20 × lens; scale bar represents 100 μm). *C* and *D*, media and lumen diameters in arterial cross-sections (n = 4). *E*, the media-to-lumen (M:L) ratio was significantly higher in cerebral arteries from the 12-week-old SHR than WKY. Two-tailed Student’s *t* test. ∗, *p* < 0.05. WKY = *blue*, SHR = *orange*. SHR, spontaneously hypertensive rat; WKY, Wistar Kyoto rat.

The basilar artery was dissected in a subset of 12-week-old rats (n = 4) and stained with Sirius red to investigate the arterial morphology at a macroscopic level (Fig. 1, *A* and *B*). This confirmed a significantly increased media diameter (Fig. 1*C*) and a reduced lumen diameter (Fig. 1*D*), accompanied with significantly increased media-to-lumen ratio in the 12-week-old SHRs compared to WKY controls (Fig. 1*E*).

### Cerebral arteries in hypertensive rats show distinct proteomic profile compared to normotensive control

**Figure 2:**
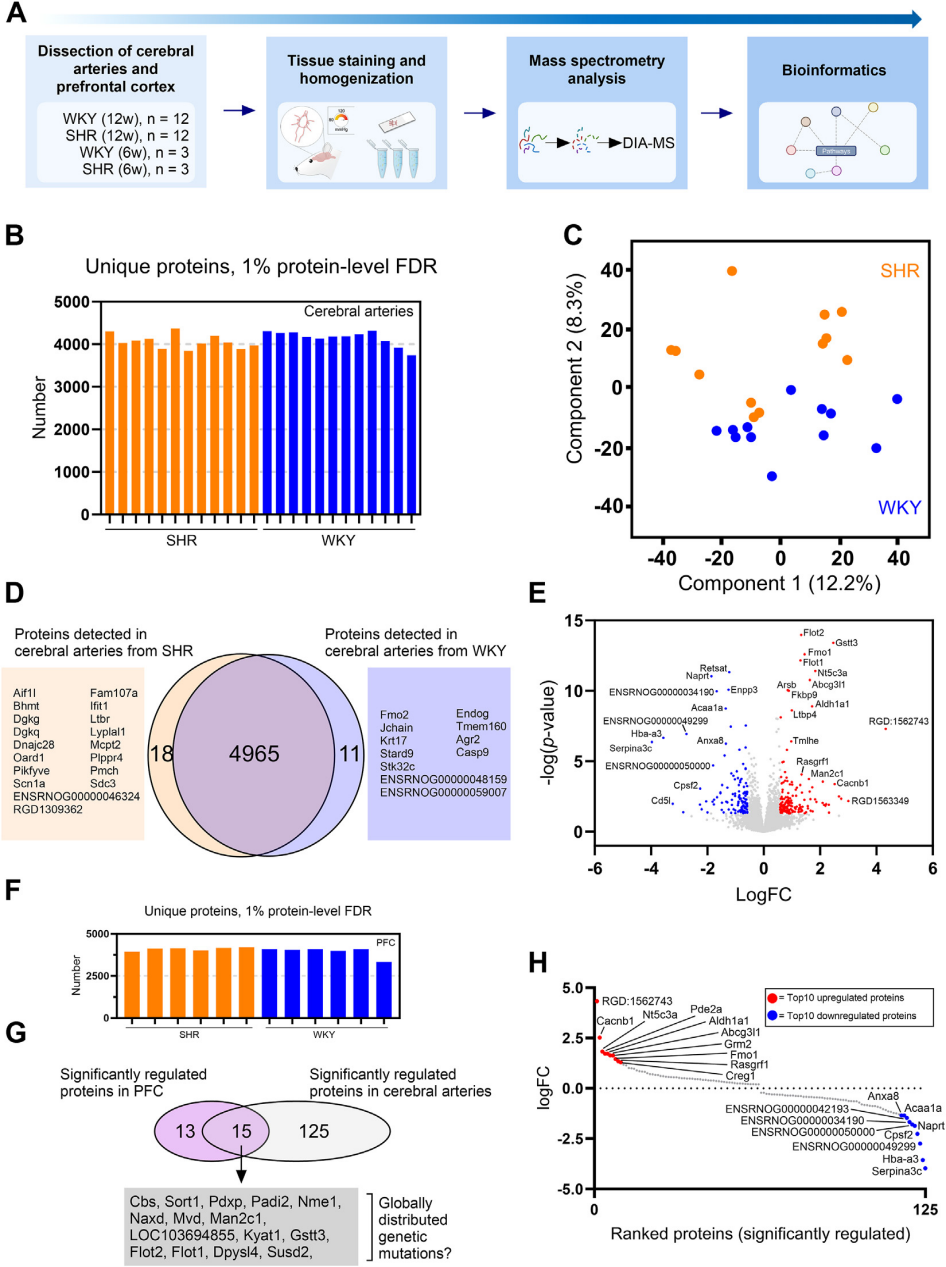
**Identification of significantly regulated proteins in cerebral arteries from hypertensive rats.***A*, study overview of the proteomic analysis. Cerebral arteries were dissected from two cohorts of Wistar Kyoto (WKY) and spontaneously hypertensive rats (SHRs) at 6 weeks (n = 3) and 12 weeks of age (n = 12). A segment of the prefrontal cortex (PFC) was extracted from the 12-week-old rats (n = 6). Extracted tissue was snap frozen and analyzed by histological staining or mass spectrometry (MS) analysis. Digested samples for MS analysis were analyzed by liquid chromatography–tandem MS (LC-MS/MS) using data-independent acquisition (DIA). Protein identification and quantification was obtained using DIA-NN software. *B*, stacked bar representation of unique proteins identified by DIA-MS across cerebral artery samples from 12-week-old SHR (*orange*) and WKY (*blue*). *C*, principal component analysis (PCA) plot of log2-transformed intensities associated with the 12-week-old rat samples. Components 1 and 2 are presented. *D*, Venn diagram showing total number of exclusive and shared proteins identified in cerebral arteries from 12-week-old SHR and WKY (*orange* and *blue circle*, respectively). *E*, volcano plot comparing protein abundance in cerebral arteries from 12-week-old SHR and WKY controls. Log2 fold change (logFC) difference and nonadjusted *p*-value are presented in volcano plot. *Red* = upregulated and *blue* = downregulated in SHR compared to WKY. Exclusive proteins are not included. *F*, stacked bar representation of unique proteins identified by DIA-MS across medial PFC samples from 12-week-old SHR (*orange*) and WKY (*blue*). *G*, Venn diagram representing number of significantly regulated proteins in statistical analysis of cerebral arteries and PFC from 12-week-old SHR and WKY rats. The number of shared proteins (n = 15) were subsequently removed from the cerebral artery list because of a possible global genetic involvement. *H*, significantly regulated proteins identified in (*G*) ranked based on logFC. *Red circle* = top10 significantly upregulated, *blue circle* = top10 significantly downregulated proteins. FDR = false-discovery rate; SHR, spontaneously hypertensive rat; WKY, Wistar Kyoto rat.

After confirming a structural difference in cerebral vasculature of the SHR compared to the WKY, we investigated the protein composition by label-free data-independent acquisition (DIA)-MS quantification. We analyzed SHR and WKY cerebral arteries at 12 weeks of age (n = 12) to capture the protein changes occurring in the early-onset hypertensive state (Fig. 2*A*). We identified a total of 4965 unique proteins that were observed across the cerebral artery samples, supporting the sensitivity of the DIA-MS approach with high reproducibility between samples (Fig. 2, *B* and *D*). Using unbiased principal component analysis (PCA), we observed two clusters corresponding to phenotype (SHR and WKY) along component 2, accounting for 8.3% variation (Fig. 2*C*).

Using a linear model (modified Student’s *t* test) (15), we identified 168 upregulated and 121 downregulated proteins (volcano plot visualization in Fig. 2*E*). After correcting *p*-values for multiple comparisons, we reduced the list to 140 proteins (73 upregulated and 67 downregulated) that displayed significantly different expression levels between the cerebral arteries from SHRs and WKY controls.

The SHR was derived from the WKY and inbred to perpetuate the hypertensive phenotype. The elevated blood pressure is therefore based on several mutations (6), many of which are likely to be unknown. To limit the influence of this factor, we performed an additional DIA-MS analysis of brain tissue samples from the medial prefrontal cortex (PFC) region of SHRs and WKY controls and observed a similar unique protein identification (∼4000 proteins per sample) (Fig. 2*F*). Using a linear regression model, we identified 28 significantly regulated proteins between SHR and WKY controls in the PFC region. Of these, 15 proteins were also present in the analysis of cerebral arteries (Fig. 2*G*). These overlapping proteins may be caused by genetic mutations with a global distribution affecting different tissues in the SHR model, which is not specific to the vasculature. Based on the scope of this study, we removed the 15 overlapping proteins, which left us with 125 significantly regulated proteins in the cerebral arteries from SHRs compared to WKY controls (Table 1 and Fig. 2*G*).

**Table 1:** List of significantly regulated proteins detected in cerebral arteries from hypertensive rats compared to normotensive control

Gene name	Protein description	logFC	AveExpr.	*p*-value	Adj. *p*-value
Abat	4-aminobutyrate aminotransferase, mitochondrial	0.49	17.62	1.03E-08	2.84E-06
Abcd3	ATP-binding cassette subfamily D member 3	−0.35	14.53	1.04E-03	4.06E-02
Abcg3l1	Isoform of Q4KM08, ABC transporter domain-containing protein	1.64	11.33	1.71E-11	1.06E-08
Acaa1a	3-ketoacyl-CoA thiolase A, peroxisomal	−1.35	11.69	1.80E-09	6.37E-07
Acaa2	Isoform of P13437, 3-ketoacyl-CoA thiolase, mitochondrial; 3-ketoacyl-CoA thiolase, mitochondrial	−0.50	14.91	1.11E-04	9.10E-03
Acadl	Long-chain–specific acyl-CoA dehydrogenase, mitochondrial	0.20	16.88	5.62E-04	2.54E-02
Acat1	Acetyl-CoA acetyltransferase, mitochondrial	0.28	15.39	4.29E-04	2.22E-02
Aco2	Aconitate hydratase, mitochondrial	0.20	17.28	9.98E-04	3.96E-02
Acp1	Low molecular weight phosphotyrosine protein phosphatase; low molecular weight phosphotyrosine protein phosphatase	0.35	14.23	1.92E-04	1.30E-02
Acss1	Acetyl-coenzyme A synthetase	0.52	12.47	5.14E-08	1.16E-05
Agpat5	PlsC domain-containing protein	−0.42	14.07	8.28E-05	7.21E-03
Aif1	Allograft inflammatory factor 1	−0.63	11.38	3.60E-04	1.94E-02
Ak3	GTP:AMP phosphotransferase AK3, mitochondrial; isoform of P29411, GTP:AMP phosphotransferase AK3, mitochondrial	−0.22	14.09	7.33E-04	3.08E-02
Aldh18a1	Delta-1-pyrroline-5-carboxylate synthase	0.60	14.23	7.76E-09	2.41E-06
Aldh1a1	Aldehyde dehydrogenase, mitochondrial; retinal dehydrogenase 1	1.72	14.29	1.27E-09	4.84E-07
Alg2	GDP-Man:Man (1)GlcNAc (2)-PP-Dol alpha-1,3-mannosyltransferase	−0.35	12.24	2.18E-04	1.44E-02
Alpl	Alkaline phosphatase, tissue-nonspecific isozyme	−0.70	14.01	6.61E-04	2.88E-02
Ampd3	AMP deaminase 3; AMP deaminase 3	0.42	12.39	6.20E-04	2.73E-02
Anxa1	Annexin A1	−0.31	16.01	6.88E-05	6.45E-03
Anxa5	Annexin A5; isoform of P14668, annexin	−0.34	19.34	5.92E-06	8.40E-04
Anxa8	Annexin A8	−1.34	10.77	5.60E-07	9.93E-05
Arsb	Arylsulfatase B	0.85	13.45	9.02E-11	4.41E-08
Aspa	Aspartoacylase	−0.65	14.52	3.53E-05	3.65E-03
Atp5mg	ATP synthase subunit g, mitochondrial	0.38	15.24	1.28E-03	4.67E-02
Cacna2d1	Isoform of P54290, VWFA domain-containing protein; voltage-dependent calcium channel subunit alpha-2/delta-1	0.42	11.93	7.79E-04	3.20E-02
Cacnb1	Voltage-dependent L-type calcium channel subunit beta-4; voltage-dependent L-type calcium channel subunit beta-4; voltage-dependent L-type calcium channel subunit beta-1	2.52	7.80	4.21E-04	2.20E-02
Cap1	Adenylyl cyclase-associated protein 2; adenylyl cyclase-associated protein 1	0.22	17.64	1.12E-04	9.10E-03
Cat	Catalase	0.46	16.32	3.27E-04	1.81E-02
Cd200	Isoform of P04218, Ig-like domain-containing protein; OX-2 membrane glycoprotein	0.50	12.32	4.92E-04	2.33E-02
Clybl	Citramalyl-CoA lyase, mitochondrial	0.49	11.13	4.10E-04	2.17E-02
Col12a1	Isoform of P70560, collagen alpha-1(XII) chain; collagen alpha-1(XII) chain	0.32	14.25	1.29E-03	4.67E-02
Col18a1	LAM_G_DOMAIN domain-containing protein	0.56	13.70	1.60E-04	1.15E-02
Cpsf2	Cleavage and polyadenylation specificity factor subunit 2	−2.26	8.60	8.83E-04	3.57E-02
Creg1	Cellular repressor of E1A-stimulated genes (predicted), isoform CRA_b	1.29	9.47	4.81E-04	2.33E-02
Cs	Isoform of Q8VHF5, citrate synthase; citrate synthase, mitochondrial	0.24	16.90	4.69E-04	2.33E-02
Ctss	Cathepsin S	−0.59	12.82	5.80E-04	2.59E-02
Cyb5r2	NADH-cytochrome b5 reductase 2	−0.83	11.06	2.23E-04	1.44E-02
Decr1	2,4-dienoyl-CoA reductase, mitochondrial	0.75	14.29	5.80E-05	5.54E-03
Dguok	Isoform of D3ZDE4, dNK domain-containing protein	0.55	14.09	8.99E-07	1.54E-04
Dhrs1	Dehydrogenase/reductase (SDR family) member 1	−0.38	12.95	2.20E-04	1.44E-02
Dnaja3	DnaJ heat shock protein family (Hsp40) member A3	0.39	12.12	6.68E-04	2.88E-02
ENSRNOG00000024757	Uncharacterized protein	−0.53	14.27	5.22E-05	5.08E-03
ENSRNOG00000034190	Isoform of F1LPW0, immunoglobulin heavy constant mu; isoform of F1LPW0, immunoglobulin heavy constant mu; isoform of F1LPW0, immunoglobulin heavy constant mu	−1.67	13.42	1.08E-10	4.48E-08
ENSRNOG00000042193	Ig-like domain-containing protein	−1.47	11.40	4.92E-04	2.33E-02
ENSRNOG00000049299	Ig-like domain-containing protein	−2.74	9.63	1.14E-07	2.37E-05
ENSRNOG00000050000	Ig lambda-2 chain C region	−1.80	10.27	2.00E-05	2.25E-03
Echs1	Enoyl-CoA hydratase, mitochondrial	0.25	16.67	6.92E-06	9.29E-04
Emb	Embigin	0.48	13.55	4.39E-04	2.25E-02
Enpp3	Ectonucleotide pyrophosphatase/phosphodiesterase family member 3	−1.25	10.75	8.36E-11	4.41E-08
Fbln5	Fibulin-5	0.56	15.12	1.33E-05	1.64E-03
Fkbp9	Peptidyl-prolyl cis-trans isomerase FKBP9	0.89	13.35	9.77E-11	4.41E-08
Flnb	Filamin B; filamin B	−0.22	15.27	3.20E-04	1.81E-02
Fmo1	Dimethylaniline monooxygenase [N-oxide-forming] 1	1.45	13.51	2.56E-13	4.24E-10
Fmo3	Dimethylaniline monooxygenase [N-oxide-forming] 4; dimethylaniline monooxygenase [N-oxide-forming] 3	−0.88	10.15	4.85E-04	2.33E-02
Gbp2	Guanylate-binding protein 1	−0.58	10.91	7.07E-05	6.45E-03
Glud1	Glutamate dehydrogenase 1, mitochondrial	0.46	17.54	8.11E-08	1.75E-05
Glul	Glutamine synthetase	−0.43	16.82	5.45E-04	2.50E-02
Grm2	Metabotropic glutamate receptor 2; metabotropic glutamate receptor 2	1.63	9.65	1.40E-03	4.97E-02
Hba-a3	GLOBIN domain-containing protein	−3.56	15.76	2.21E-07	4.39E-05
Hdhd2	Haloacid dehalogenase-like hydrolase domain-containing protein 2; isoform of Q6AYR6, immediate early response 3-interacting protein 1	1.18	11.23	7.42E-04	3.10E-02
Hexb	Beta-hexosaminidase subunit beta	0.29	13.41	1.25E-03	4.67E-02
Hint3	Histidine triad nucleotide-binding protein 3	0.62	11.88	1.30E-04	1.01E-02
Hmgcs2	Hydroxymethylglutaryl-CoA synthase, cytoplasmic; hydroxymethylglutaryl-CoA synthase, mitochondrial	0.50	12.77	1.16E-03	4.42E-02
Hsd17b11	Estradiol 17-beta-dehydrogenase 11	0.55	11.83	2.62E-04	1.61E-02
Hspd1	60 kDa heat shock protein, mitochondrial	0.21	16.27	9.97E-04	3.96E-02
Iba57	Iron-sulfur cluster assembly factor IBA57	0.36	13.05	8.84E-04	3.57E-02
Idh1	Isocitrate dehydrogenase [NADP] cytoplasmic; isocitrate dehydrogenase [NADP] cytoplasmic	−0.30	14.38	4.77E-04	2.33E-02
Igf2	Insulin-like growth factor II	−0.35	14.50	2.25E-05	2.43E-03
Igg-2a	Ig gamma-2A chain C region	−1.03	14.16	1.91E-04	1.30E-02
Igh-1a	Ig gamma-2B chain C region	−1.15	14.28	7.27E-05	6.45E-03
Igtp	Interferon gamma-induced GTPase	−0.43	13.51	3.05E-04	1.76E-02
Irgm	Immunity-related GTPase family M protein	−0.42	12.90	1.73E-05	2.00E-03
Isg15	ISG15 ubiquitin-like modifier	−0.84	12.48	3.03E-04	1.76E-02
Isyna1	Inositol-3-phosphate synthase 1	−0.50	14.48	1.44E-04	1.10E-02
Itgb2	Integrin beta	−0.39	12.65	6.72E-04	2.88E-02
Itgb4	Isoform of Q64632, integrin beta; integrin beta-4	−0.57	10.76	1.96E-04	1.32E-02
Ivd	Isovaleryl-CoA dehydrogenase, mitochondrial	0.22	15.15	1.17E-03	4.42E-02
Kank2	KN motif and ankyrin repeat domain-containing protein 2	−0.32	14.18	1.03E-03	4.05E-02
Krt8	Peripherin; vimentin; glial fibrillary acidic protein; glial fibrillary acidic protein; keratin, type II cytoskeletal 8; keratin, type II cytoskeletal 8; keratin, type II cytoskeletal 8; keratin, type II cytoskeletal 8; keratin, type II cytoskeletal 8	−0.88	12.58	1.36E-03	4.87E-02
Lgals3	Galectin-3	−0.52	13.76	2.81E-06	4.23E-04
Lss	Lanosterol synthase	−1.17	10.29	3.48E-08	8.65E-06
Ltbp4	Isoform of D4A917, latent-transforming growth factor beta-binding protein 4	0.99	12.95	2.44E-09	8.08E-07
Mfge8	Lactadherin	0.70	14.67	1.07E-05	1.40E-03
Mrc1	Mannose receptor, C type 1	−0.64	12.28	3.16E-05	3.34E-03
Mvp	Major vault protein	−0.28	13.86	1.31E-03	4.70E-02
Naprt	Nicotinate phosphoribosyltransferase	−1.86	11.08	9.21E-12	6.54E-09
Ndufv3	Isoform of Q6PCU8, complex I-9kD; NADH dehydrogenase [ubiquinone] flavoprotein 3, mitochondrial	0.45	16.49	9.34E-09	2.73E-06
Nit1	Deaminated glutathione amidase	0.40	13.45	1.21E-03	4.56E-02
Nqo1	NAD(P)H dehydrogenase [quinone] 1	−0.64	13.62	1.06E-06	1.75E-04
Nt5c3a	5′-nucleotidase	1.83	12.85	3.89E-12	3.86E-09
Nup107	Nuclear pore complex protein Nup107	−1.29	8.78	7.54E-04	3.12E-02
Oas1a	Isoform of Q05961, 2′-5′ oligoadenylate synthase; 2′-5′-oligoadenylate synthase 1A	−0.79	12.12	1.77E-04	1.26E-02
Pacsin2	Isoform of Q9QY17, isoform 2 of PKC and casein kinase substrate in neurons 2 protein; isoform of Q9QY17, isoform 2 of PKC and casein kinase substrate in neurons 2 protein	−0.32	13.94	1.51E-04	1.12E-02
Pcca	Isoform of P14882, propanoyl-CoA:carbon dioxide ligase subunit alpha; propionyl-CoA carboxylase alpha chain, mitochondrial	0.20	15.31	5.55E-04	2.53E-02
Pccb	Propionyl-CoA carboxylase beta chain, mitochondrial; isoform of P07633, propionyl coenzyme A carboxylase, beta polypeptide	0.26	15.65	1.29E-04	1.01E-02
Pcdh7	Protocadherin 7	1.13	8.83	1.06E-03	4.06E-02
Pde2a	cGMP-dependent 3′,5′-cyclic phosphodiesterase	1.72	12.20	7.02E-04	2.98E-02
Pfkl	ATP-dependent 6-phosphofructokinase, liver type; ATP-dependent 6-phosphofructokinase, liver type; ATP-dependent 6-phosphofructokinase, liver type; ATP-dependent 6-phosphofructokinase, liver type	0.66	11.99	1.19E-05	1.52E-03
Pgm5	Phosphoglucomutase 5	0.29	14.50	1.29E-03	4.67E-02
Phgdh	D-3-phosphoglycerate dehydrogenase	0.82	14.14	1.56E-06	2.43E-04
Plec	Isoform of P30427, Calponin-homology (CH) domain-containing protein; isoform of P30427, isoform 2 of Plectin	−0.30	15.18	2.55E-04	1.60E-02
Podxl	Podocalyxin	−0.45	13.45	1.28E-03	4.67E-02
Poglut3	Protein O-glucosyltransferase 3	0.58	12.30	4.08E-05	4.13E-03
Ptpru	Isoform of F1MAG7, protein-tyrosine-phosphatase	−0.92	10.73	1.55E-04	1.13E-02
RGD1306556	Tankyrase_bdg_C domain-containing protein	0.84	11.63	2.58E-04	1.60E-02
RGD:1562743	Ig kappa chain C region, B allele; Ig kappa chain C region, A allele	4.33	11.88	5.00E-08	1.16E-05
Rasgrf1	Ras-specific guanine nucleotide-releasing factor 1; Ras-specific guanine nucleotide-releasing factor 1	1.34	10.32	8.59E-05	7.35E-03
Retsat	Isoform of Q8VHE9, All-trans-13,14-dihydroretinol saturase, isoform CRA_b; All-trans-retinol 13,14-reductase	−1.22	13.04	4.72E-12	3.90E-09
Rtn4ip1	PKS_ER domain-containing protein	0.70	11.59	3.12E-04	1.78E-02
Rtp4	zf-3CxxC domain-containing protein	−1.08	10.22	1.08E-04	9.10E-03
Septin2	Septin-2	−0.27	14.75	1.65E-05	1.95E-03
Serpina3c	SERPIN domain-containing protein	−3.97	11.32	4.30E-07	7.91E-05
Serpina6	Corticosteroid-binding globulin	−0.86	13.06	1.53E-06	2.43E-04
Shmt2	Serine hydroxymethyltransferase	−0.45	12.89	6.87E-06	9.29E-04
Slc14a1	Urea transporter 1	1.01	10.98	4.64E-04	2.32E-02
Sparcl1	SPARC-like protein 1	0.34	13.82	7.27E-05	6.45E-03
Stab1	Stabilin 1	−0.47	11.87	3.46E-04	1.89E-02
Supt5h	Transcription elongation factor SPT5	−0.55	12.89	6.17E-04	2.73E-02
Tapbp	Isoform of Q99JC6, TAP-binding protein (Fragment); Ig-like domain-containing protein	−0.96	14.54	5.18E-05	5.08E-03
Tecr	Very-long-chain enoyl-CoA reductase	−0.46	13.36	3.25E-04	1.81E-02
Tjp2	Tight junction protein 2	−0.50	12.35	5.34E-04	2.48E-02
Tmlhe	Trimethyllysine dioxygenase, mitochondrial	0.97	11.41	3.94E-07	7.52E-05
Ttyh1	Protein tweety homolog 1	0.67	12.90	1.05E-03	4.06E-02
Txndc5	RCG43947	0.24	14.10	5.02E-04	2.35E-02
Ufl1	E3 UFM1-protein ligase 1	−0.39	11.89	2.95E-04	1.76E-02

Description of the significantly regulated proteins identified when comparing the proteome of 12-week-old cerebral arteries from spontaneously hypertensive rats (SHR) and normotensive Wistar Kyoto rats (WKY). logFC = log2 fold change difference. AveExpr. = average expression. SHR *versus* WKY comparison.

We ranked the top 10 upregulated and downregulated proteins based on the log2-transformed differences (Table 1 and Fig. 2*H*). Examples of downregulated proteins included Serpina3c, Naprt, and Cpsf2. These proteins play key roles in protease inhibition and oxidative stress control. Examples of upregulated proteins included Cacnb1 and Pde2a. These proteins play key roles in immune activation and promotion of oxidative stress.

### Pathway analysis identifies mechanistic differences in hypertensive rats compared to normotensive control

**Figure 3:**
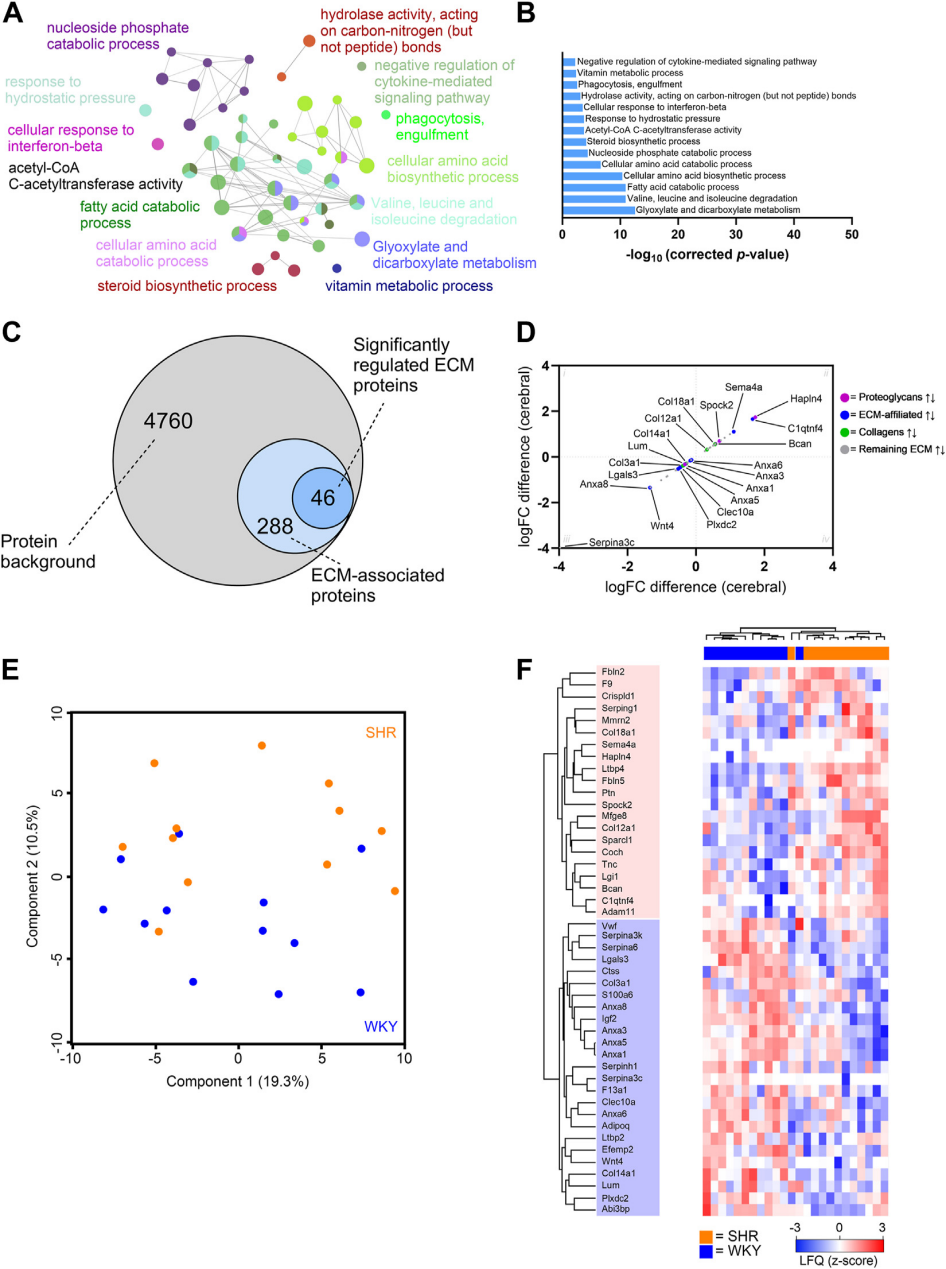
**Pathway and clustering analysis of proteins identified in cerebral arteries from hypertensive rats.***A*, gene overrepresentation analysis of significantly regulated proteins (n = 125) identified from the spontaneously hypertensive rats (SHR) *versus* normotensive Wistar Kyoto rats (WKY) comparison using ClueGO. The size of each node is based on a significance score, and each cluster of related terms is labeled with the highest significance score of annotated ontology reference sets after *p*-value correction. *B*, significance score identified in enrichment analysis of regulated proteins in SHR compared to WKY. *C*, Venn diagram showing the number of extracellular matrix (ECM)–associated proteins in dataset using an ECM database (7, 16, 17). *D*, scatter plot of log2 fold changes (logFC) for all significantly regulated ECM-associated proteins (moderated *t* test; non-adj. *p*-values are used). Each protein is colored as follows: (proteoglycan = *purple*, ECM-affiliated = *blue*, collagens = *green*, remaining ECM = *gray*). *Black arrows* indicate direction of change. *E*, principal component analysis (PCA) of ECM-associated proteins in cerebral arteries. Components 1 and 2 are presented. *F*, unsupervised hierarchical clustering of 46 significantly regulated ECM-associated proteins identified in (*C*). Z-scored label-free quantification (LFQ) values from cerebral artery samples are depicted. SHR = *orange*, WKY = *blue*.

We investigated whether particular pathways were regulated in the SHR compared to the WKY control. Using gene overrepresentation analysis, we identified 14 clusters of related Gene Ontology (GO) and Kyoto Encyclopedia of Genes and Genomes (KEGG) terms (Fig. 3*A* and Table 2). The clusters with lowest group term significance score were “Glyoxylate and dicarboxylate metabolism”, “Valine, leucine and isoleucine degradation”, and “fatty acid catabolic process” (Fig. 3*B*).

**Table 2:** Pathway analysis of proteins associated with hypertension

Term	Ontology source	Term *p*-value corrected with Bonferroni step down	Group *p*-value corrected with Bonferroni step down	% Associated genes	Associated genes found
Negative regulation of cytokine-mediated signaling pathway	GO BiologicalProcess	6.18E-03	6.18E-03	4.7	Dnaja3, Isg15, Oas1a
Phagocytosis, engulfment	GO BiologicalProcess	1.10E-02	2.75E-03	5.4	Aif1, Igh-6, Itgb2, Mfge8
Response to hydrostatic pressure	GO BiologicalProcess	8.57E-04	1.84E-04	27.3	Col18a1, Krt8, Plec
Vitamin metabolic process	GO BiologicalProcess	1.15E-02	4.61E-03	4.2	Alpl, Clybl, Nqo1, Shmt2
Cellular response to interferon-beta	GO BiologicalProcess	1.43E-03	3.10E-04	10.8	Gbp2, Igtp, Irgm, Oas1a
Hydrolase activity, acting on carbon-nitrogen (but not peptide) bonds	GO MolecularFunction	3.62E-03	7.61E-04	4.2	Ampd3, Aspa, Cat, Cd200, Hint3, Nit1
Cellular amino acid catabolic process	GO BiologicalProcess	9.68E-05	2.42E-07	6.6	Abat, Acat1, Glud1, Ivd, Pcca, Pccb, Shmt2
Steroid biosynthetic process	GO BiologicalProcess	1.38E-03	8.03E-05	4.2	Abcd3, Cyb5r2, Hmgcs2, Hsd17b11, Igf2, Lss, Tecr
Acetyl-CoA C-acetyltransferase activity	GO MolecularFunction	2.11E-04	1.81E-04	42.9	Acaa1a, Acaa2, Acat1
Glyoxylate and dicarboxylate metabolism	KEGG	4.53E-12	2.88E-13	29.0	Acat1, Aco2, Acss1, Cat, Cs, Glul, Pcca, Pccb, Shmt2
Nucleoside phosphate catabolic process	GO BiologicalProcess	1.82E-03	4.21E-05	6.5	Acat1, Ampd3, Enpp3, Nt5c3a, Pde2a
Cellular amino acid biosynthetic process	GO BiologicalProcess	1.19E-05	4.52E-11	9.1	Abat, Aldh18a1, Aldh1a1, Glud1, Glul, Phgdh, Shmt2
Valine, leucine, and isoleucine degradation	KEGG	1.47E-09	1.21E-11	16.1	Abat, Acaa1a, Acaa2, Acat1, Echs1, Hmgcs2, Ivd, Pcca, Pccb
Fatty acid catabolic process	GO BiologicalProcess	2.19E-09	1.21E-11	9.8	Abcd3, Acaa1a, Acaa2, Acadl, Acat1, Aldh1a1, Decr1, Echs1, Ivd, Pcca, Pccb

ClueGO-enrichment analysis of significantly regulated proteins identified in cerebral arteries from the comparison of 12-week-old spontaneously hypertensive rats (SHR) and normotensive Wistar Kyoto rats (WKY). The annotations represent related Gene Ontology (GO) or Kyoto Encyclopedia of Genes and Genomes (KEGG) terms enriched as nodes. The term represents the highest significance score.

Previously, we identified significant changes in the ECM-associated proteins in mesenteric arteries from 12-week-old SHRs (7). Based on this previous finding, we enriched our cerebral artery data for ECM proteins using the same ECM database (7, 16, 17). We identified 334 ECM-associated protein in our total dataset, 46 of which were regulated significantly between SHR and WKY controls (non-adj. *p*-value; Fig. 3, *C* and *D*). However, using PCA, we were not able to separate SHR and WKY phenotype samples from each other (Fig. 3*E*). Similarly, when applying unsupervised hierarchical clustering analysis of significantly regulated ECM proteins, we did not observe a clear separation of SHR and WKY control samples (z-score normalized; Fig. 3*F*), suggesting that the ECM proteins alone were not able to differentiate the two groups.

**Figure 4:**
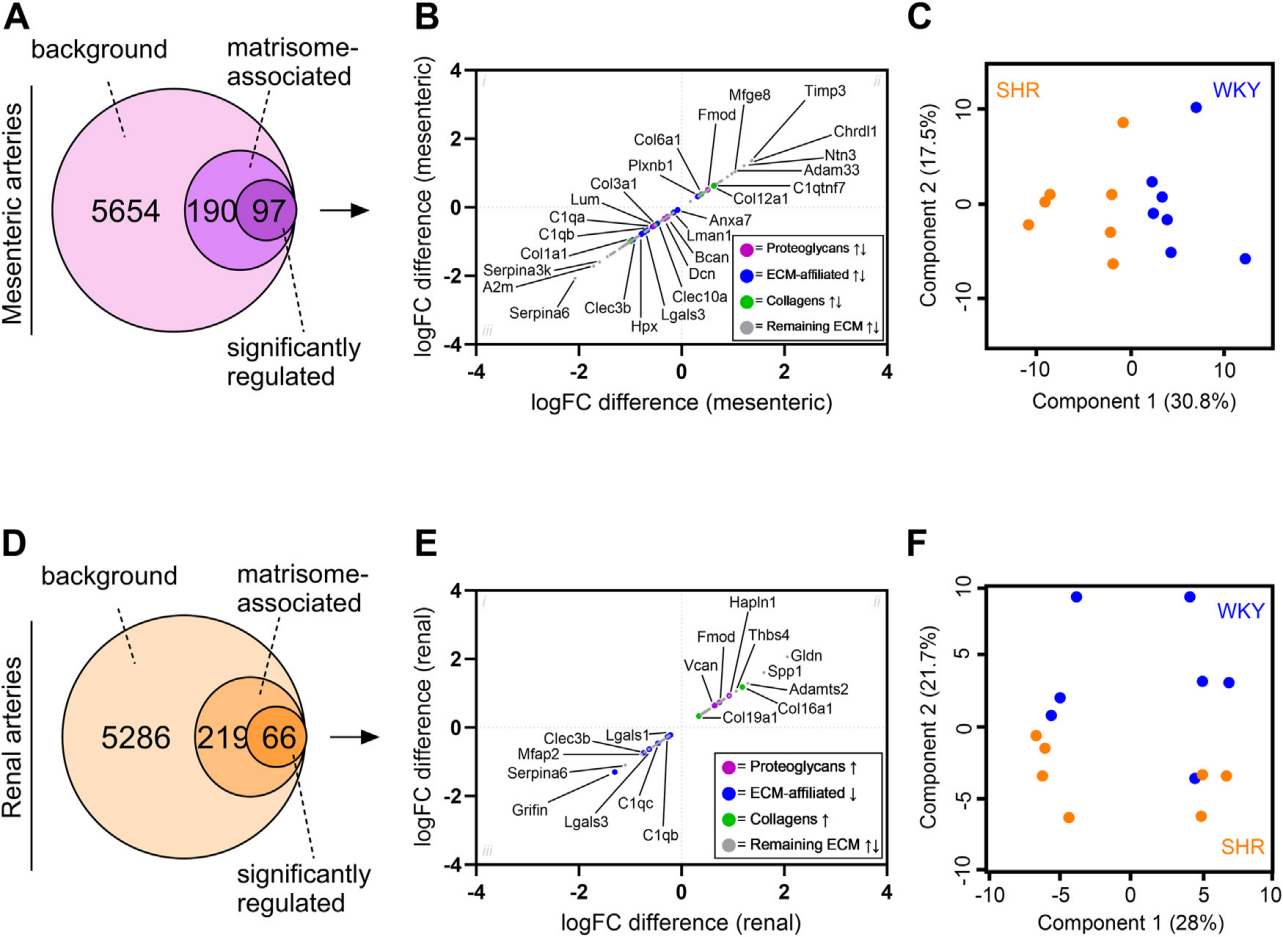
**Extracellular matrix profiling in two vascular beds from early-onset hypertensive rats.***A* and *D*, Venn diagrams representing the number of ECM-associated proteins in mesenteric and renal artery datasets from a previous publication (7). The annotations were based on the “matrisome” database (7, 16, 17). *B* and *E*, scatter plot of log2 fold changes (logFC) for all significantly regulated ECM-associated proteins when comparing proteomic profile of 12-week-old spontaneously hypertensive rats (SHR) and normotensive Wistar Kyoto rats (WKY) (*t* test; non-adj. *p*-values are used). Each protein is colored as (proteoglycan = *purple*, ECM-affiliated = *blue*, collagens = *green*, remaining ECM = *gray*). *Black arrows* indicate direction of change. *C* and *F*, principal component analysis (PCA) of ECM-associated proteins in mesenteric and renal artery datasets, respectively. *Orange* = SHR, *blue* = WKY control. Components 1 and 2 are presented. ECM, extracellular matrix.

To elucidate the ECM differences across vascular beds, we applied the same analysis to our recently published database on mesenteric and renal arteries in SHRs at the early-onset stage (7). To enable this comparison we re-analyzed the data with the same analysis software as presented in this study (DIA-NN). This led to a total identification of 5941 and 5571 unique proteins in the mesenteric and renal arteries, respectively (n = 14 samples in each arterial bed type (SHR = 7 and WKY = 7)). After comparing both datasets to the ECM database and applying a *p*-value cutoff <0.05 (Student’s *t* test), we identified 97 and 66 significantly regulated ECM-associated proteins in the mesenteric and renal artery datasets, respectively (non-adj. *p*-values; Fig. 4, *A* and *D*). When creating plots encompassing protein expression differences between SHR and WKY control, we observed a general decrease of ECM-affiliated proteins in both vascular beds, while the expression of proteoglycans were mixed in mesenteric and increased in renal arteries (Fig. 4, *B* and *E*). Furthermore, when using unbiased PCA, we observed clear clustering corresponding to the SHR and WKY phenotypes in both mesenteric and renal arteries (Fig. 4, *C* and *F*). Taken together with Figure 3, these data suggest that although ECM changes do occur in SHR cerebral arteries, based on the limited group separation, the changes were not as prominent as in SHR mesenteric and renal arteries.

### Early-onset hypertensive rats display an imbalanced angiogenic profile in cerebral arteries but not in other systemic arteries

**Figure 5:**
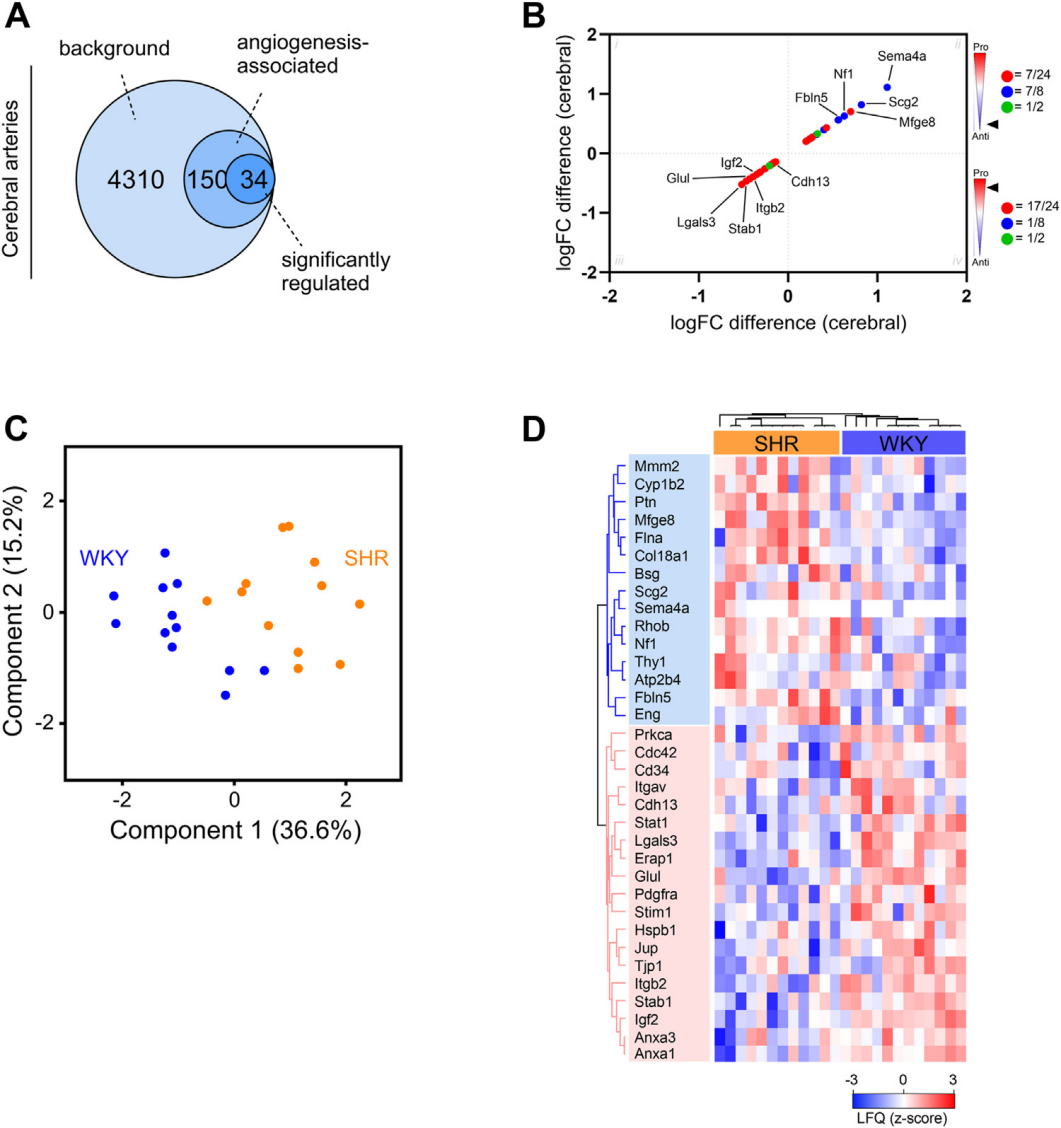
**Clustering analysis of angiogenesis-associated proteins can differentiate between cerebral arteries from hypertensive and normotensive rats.***A*, Venn diagrams representing the number of significantly regulated angiogenesis-associated proteins in cerebral artery dataset. The annotations were based on the Gene Ontology (GO) reference set (GO:0001525). *B*, scatter plot of log2 fold changes (logFC) for all significantly regulated angiogenesis-associated proteins when comparing proteomic profile of cerebral arteries from 12-week-old spontaneously hypertensive rats (SHR) and normotensive Wistar Kyoto rats (WKY) (moderated *t* test; non-adj. *p*-values were used). Each protein is colored as pro-angiogenic (=*red*), anti-angiogenic (=*blue*), and unknown or both (=*green*). *Black arrow* indicates angiogenic profile in respective squares (ii-iii). *C*, principal component analysis (PCA) plot of log2-transformed intensities associated with angiogenesis-associated proteins from 12-week-old SHR (*orange*) or WKY control (*blue*). Components 1 and 2 are presented. *D*, unsupervised hierarchical clustering of significantly regulated angiogenesis-associated proteins. Z-scored label-free quantification (LFQ) values are depicted.

Hypertension and many cerebrovascular diseases are associated with compromised vascular integrity and promotion of vascular rarefaction. To investigate the latter, we compared our total protein background in the cerebral arteries to an angiogenesis database (GO term enrichment; GO:0001525) because dysregulated angiogenesis can lead to vascular rarefaction (loss of capillaries and arterioles) (1). This enrichment identified 184 proteins related to angiogenesis, of which 34 proteins were significantly regulated (non-adj. *p*-values; Fig. 5*A*). We used the fold change difference identified from the SHR *versus* WKY comparison, to create a plot encompassing angiogenic changes (Fig. 5*B*; pro-angiogenic = red, anti-angiogenic = blue, unknown or both = green). Importantly, a clear pattern of upregulated anti-angiogenic proteins (=7/8, 87.5%) and downregulated pro-angiogenic proteins (17/24, 70.83%) was observed (Fig. 5*B* and Table S1).

Using a PCA plot based on the angiogenesis-associated proteins only, we observed two clusters corresponding to the phenotype (SHR and WKY) along component 1, accounting for 36.6% variation (Fig. 5*C*). This clustering was supported by an unsupervised hierarchical clustering analysis of significantly different angiogenesis-associated proteins that similarly showed clear grouping of SHR and WKY based on protein expression intensities (z-score normalized; Fig. 5*D*).

**Figure 6:**
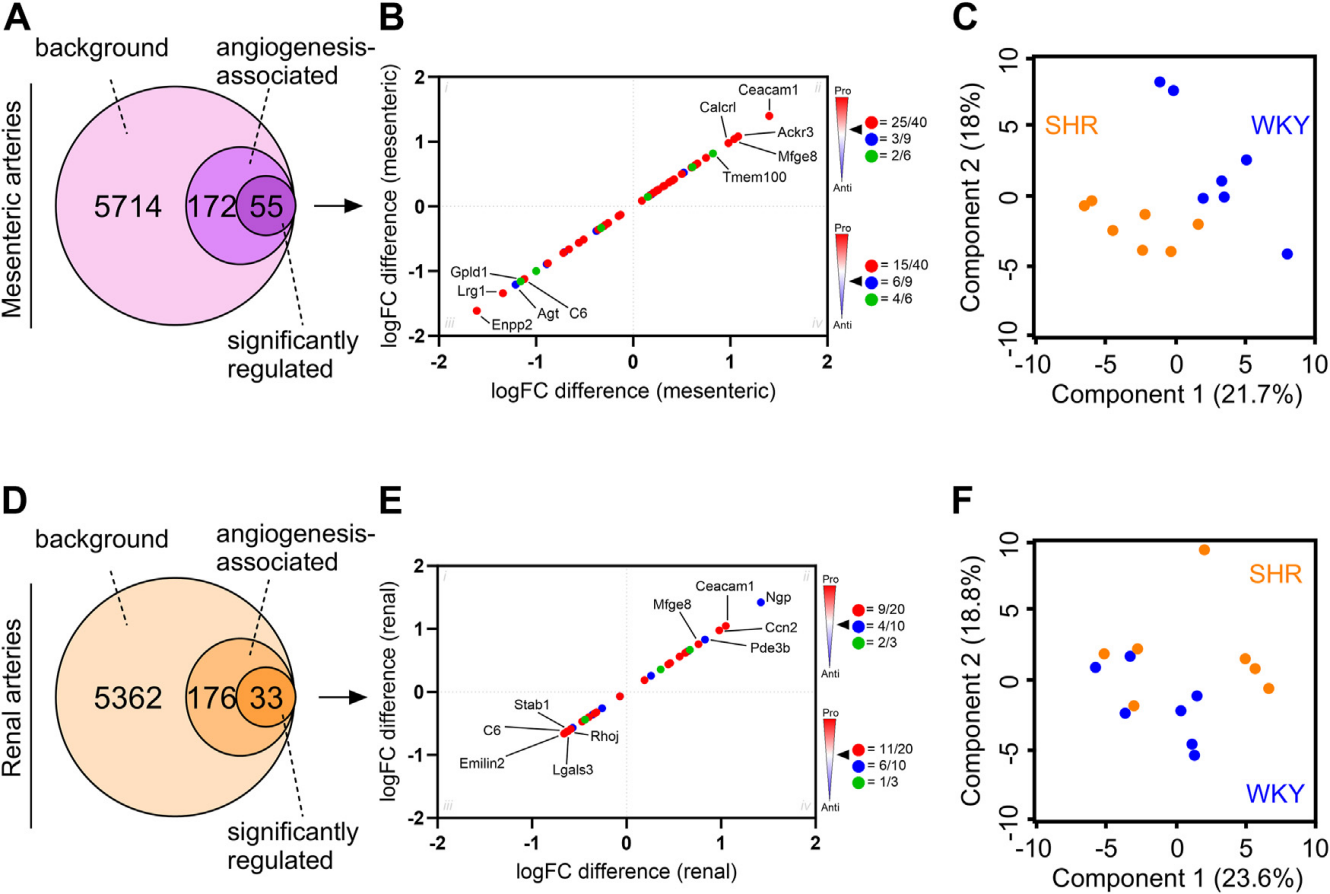
**Angiogenic profile in mesenteric and renal arteries from early-onset hypertensive rats.***A* and *E*, Venn diagrams representing the number of angiogenesis-associated proteins in the mesenteric and renal artery datasets from a previous publication (7). The annotations were based on the Gene Ontology (GO) reference set (GO:0001525). *B* and *D*, scatter plots representing fold change (logFC) differences of significantly regulated angiogenic proteins in mesenteric and renal arteries from 12-week-old spontaneously hypertensive rats (SHR) and normotensive Wistar Kyoto rats (WKY) comparisons, respectively. Each protein is colored as pro-angiogenic (=*red*), anti-angiogenic (=*blue*), and unknown or both (=*green*). *Black arrow* indicates angiogenic profile in respective squares (ii-iii). *C* and *F*, principal component analysis (PCA) plot of log2-transformed intensities associated with angiogenesis-associated proteins from mesenteric and renal artery datasets. SHR = *orange*, WKY = *blue*. Components 1 and 2 are presented.

To determine whether this angiogenic pattern was maintained in different arterial beds, we repeated the analysis to our recently published database on mesenteric and renal arteries in SHRs. After comparing both datasets to the angiogenesis database (GO term enrichment; GO:0001525) and applying a *p*-value cutoff <0.05 (Student’s *t* test), we identified 55 and 33 significantly regulated angiogenesis-associated proteins in the mesenteric and renal artery datasets, respectively (non-adj. *p*-values; Fig. 6, *A* and *D*). When creating similar plots encompassing protein expression differences, we observed that both pro- and anti-angiogenic proteins were distributed equally between upregulation and downregulation (Fig. 6, *B* and *E*, Tables S2, and S3). Furthermore, when using PCA plots based on the angiogenesis-associated proteins in both vascular beds, we did not observe clear clustering corresponding to the SHR and WKY control phenotypes in renal arteries, while a trend was observed in mesenteric arteries (Fig. 6, *C* and *F*). This suggests that the angiogenic imbalance observed in the cerebral arteries was not systemic but rather local to the cerebrovasculature.

### Two angiogenic proteins, Fbln5 and Cdh13, show opposite expression pattern across vascular beds

**Figure 7:**
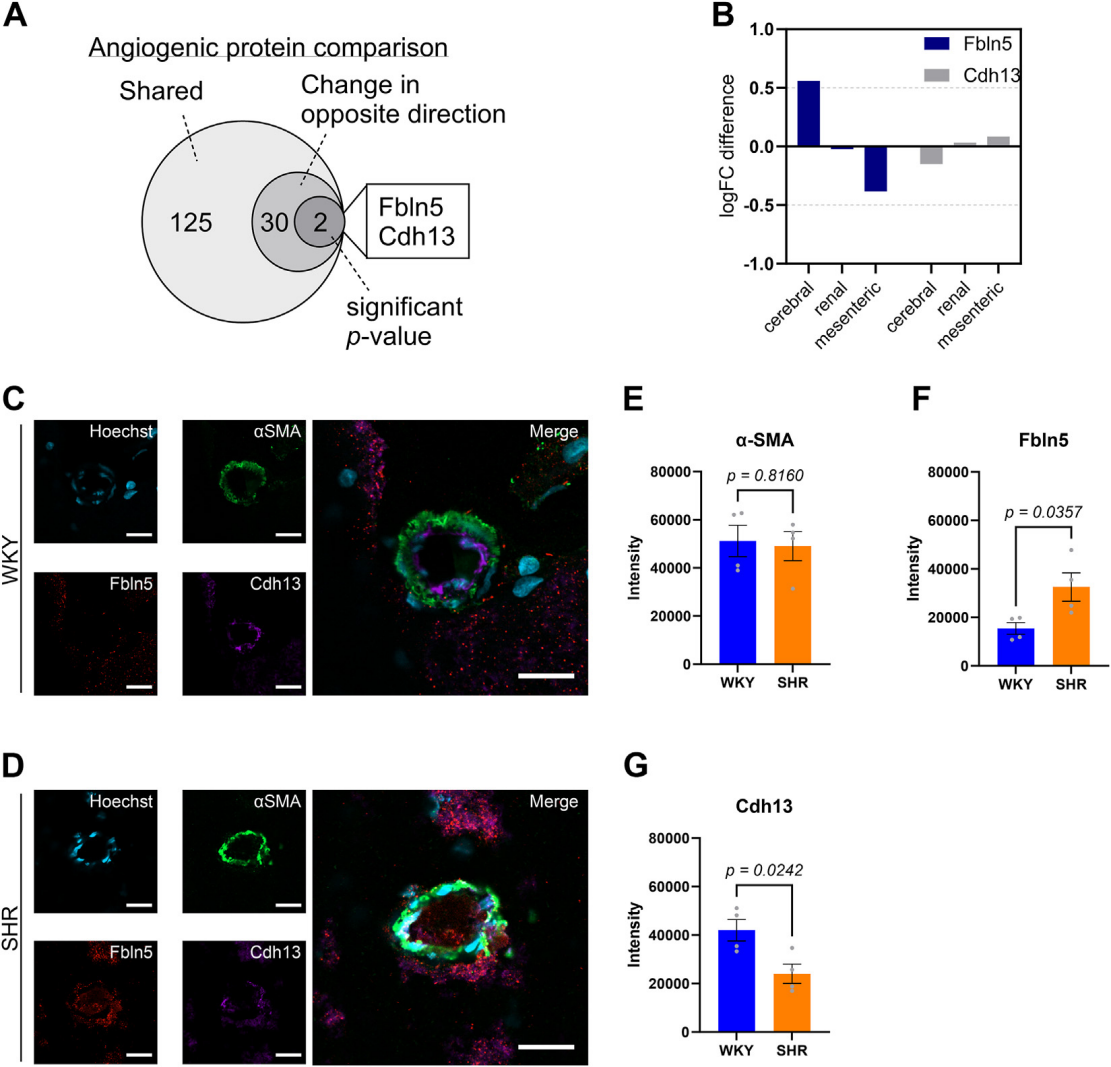
**Differential expression of two candidate proteins across vascular beds.***A*, Venn diagram showing number of angiogenesis-associated proteins in cerebral arteries that are shared with mesenteric and renal arteries but change in opposite direction. *B*, column graph depicting logFC difference of Fbln5 (=*blue*) and Cdh13 (=*gray*) in cerebral, renal, and mesenteric artery comparisons between 12-week-old spontaneously hypertensive rats (SHR) and normotensive Wistar Kyoto rats (WKY). *C*–*G*, immunohistochemistry analysis of α-SMA (*green*), Fbln5 (*red*), Cdh13 (*magenta*) in cerebral arteries from WKY controls and SHRs (n = 4 biological replicates, 2–4 technical replicates per rat). Scale bar represents 20 μm. Max fluorescent intensity was measured across a line. SEM is included on column graphs (WKY = *blue*, SHR = *orange*).

To investigate whether certain angiogenic proteins could be impacting the cerebral artery to a greater extent than the systemic arteries in the SHR, we identified angiogenic proteins that were shared between the three vascular beds (before *p*-value filtering) and focused on proteins expressed in the cerebral arteries that were changing in the opposite direction to mesenteric and renal arteries (Fig. 7*A*). This left us with 32 proteins, of which two (Fbln5 and Cdh13) were significantly regulated in the cerebral artery comparison from SHRs and WKY controls (Fig. 7*B*). Notably, Fbln5 and Cdh13 were not the most regulated angiogenic proteins in cerebral arteries; however, their opposite expression could represent a key biological mechanism that determines the cerebral artery response to increased blood pressure. To validate the MS data, we performed immunohistochemistry analysis, which showed an increase in mean fluorescent intensity of Fbln5 in cerebral arteries from the SHR compared with WKY control (*p* = 0.0357; Student’s *t* test; Fig. 7, *C*, *D*, and *F*). Furthermore, the mean fluorescent intensity for Cdh13 was reduced in SHRs (*p* = 0.0242; Student’s *t* test; Fig. 7, *C*, *D*, and *G*).

### Angiogenesis imbalance is not present in pre-hypertensive rats

**Figure 8:**
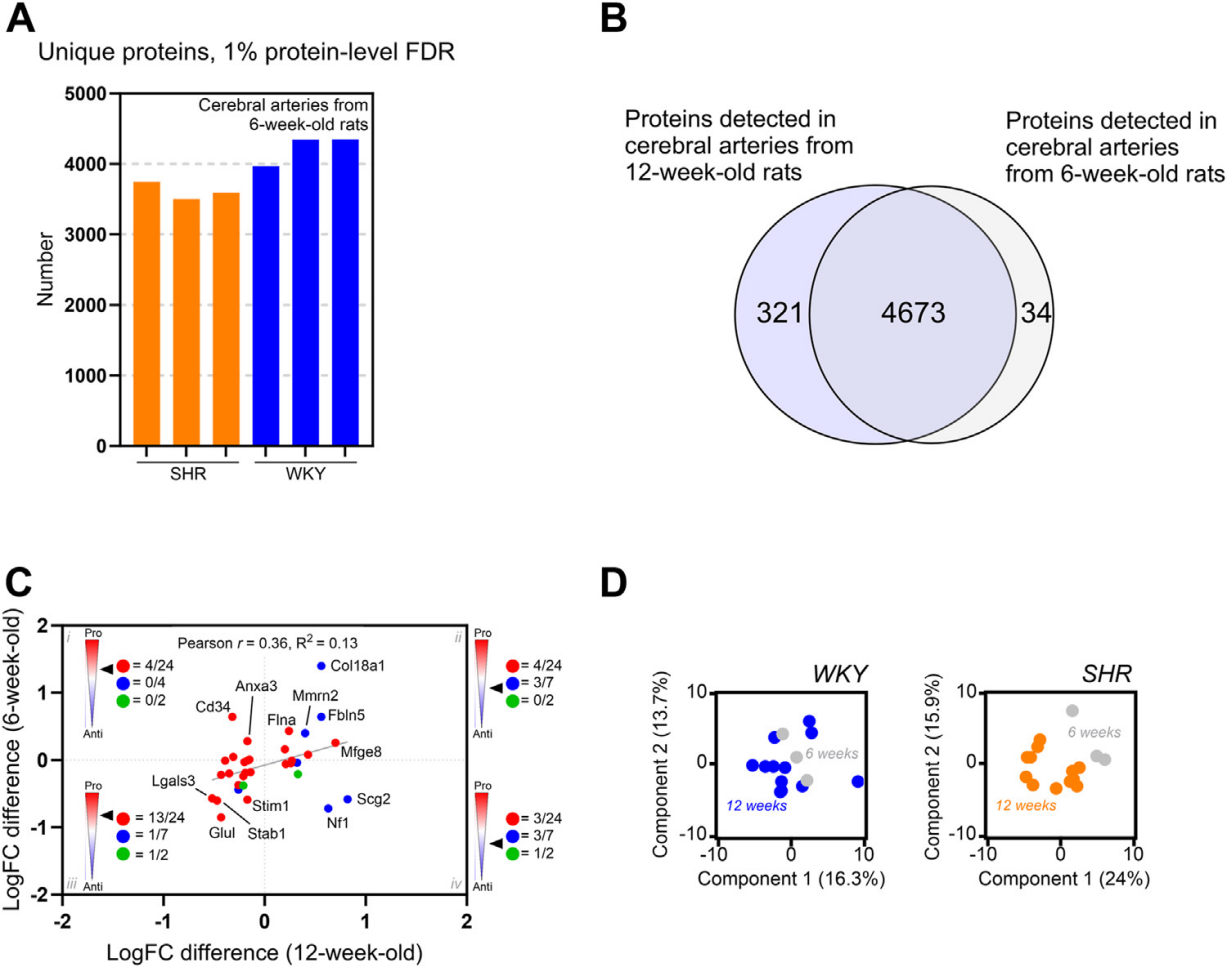
**Blood pressure–induced angiogenic imbalance in the hypertensive rat.***A*, stacked bar representation of unique proteins identified by data-independent acquisition mass spectrometry (DIA-MS) across cerebral artery samples from 6-week-old spontaneously hypertensive rats (SHR, *orange*) and normotensive Wistar Kyoto controls (WKY, *blue*). *B*, Venn diagram depicting protein overlap between 6- and 12-week-old cerebral artery datasets. *C*, scatter plot of log2 fold changes (logFC) for all significantly regulated angiogenesis-associated proteins when comparing proteomic profile of cerebral arteries from 6- and 12-week-old SHR and normotensive Wistar Kyoto rats (WKY) (x-axis = 12-week-old, y-axis = 6-week-old). Each protein is colored as pro-angiogenic (=*red*), anti-angiogenic (=*blue*), and unknown or both (=*green*). *Black arrow* indicates angiogenic profile in respective squares (i-iv). Pearson *r* coefficient (= 0.36) is indicated by *gray line*. *D*, principal component analysis (PCA) plot of log2-transformed intensities from angiogenesis-associated proteins associated with WKY samples (*left*) and SHR samples (*right*). Components 1 and 2 are presented.

We investigated the protein composition in cerebral arteries from pre-hypertensive rats (6-week-old) and age-matched WKY controls by label-free DIA-MS quantification (n = 3 in each group). Our approach led to a reproducible identification across the 6-week-old samples in both groups (Fig. 8*A*). When comparing to the 12-week-old dataset, we identified 4673 shared proteins that were observed in both datasets, supporting similar proteomic backgrounds despite the age difference (Fig. 8, *A* and *B*).

To determine whether the angiogenic changes identified in 12-week-old cerebral arteries from SHRs were also present at the pre-hypertensive state, we performed a similar angiogenic enrichment of shared proteins. We then created correlation plots using the expression difference calculated between SHR *versus* WKY at both time points (Fig. 8*C*). Both pro- and anti-angiogenic proteins were upregulated at both the 6- and 12-weeks comparisons (Fig. 8*C* and Table S4). Furthermore, the Pearson *r* and R^2^ coefficients (0.36 and 0.13, respectively; Fig. 8*C*) did not support a correlation between the two time points.

To account for age-associated changes within the WKY or SHR groups independently, we applied PCA plots on shared angiogenesis-associated proteins only (Fig. 8*D*). No clustering was observed in the 6- and 12-week-old WKY samples, suggesting that aging alone was not capable of changing the angiogenic profile. On the contrary, a clear clustering association was observed when looking at the 6- and 12-week-old SHR samples on component 1, suggesting that the angiogenic imbalance detected in the cerebral arteries was driven by elevated blood pressure in the SHR model and thus blood pressure dependent (Fig. 8*D*).

Interestingly, expression of Fbln5 was upregulated significantly in the 6-week-old SHRs compared to WKYs (logFC difference = 0.64; *p*-value = 0.033, Adj. *p*-value = 0.411), whereas the expression of Cdh13 was not different between the 6-week-old SHR and WKY control (logFC difference = 0.01; *p*-value = 0.959, Adj. *p*-value = 1).

## Discussion

Remodeling of the arterial wall in hypertension is a critical determinant of disease progression and contributes to the risk of developing cardiovascular disease. In order to identify the pathways and mechanisms underlying hypertension-related vascular remodeling, we have employed an in-depth proteomic approach in this study and in our previous publication (7). In this study, we identified several proteins with dysregulated expression in the cerebral vasculature of the SHR, early after the onset of hypertension (12-week-old). Enrichment analysis revealed that proteins involved in angiogenesis were changed in the SHR cerebral arteries in a pattern suggestive of reduced angiogenesis, which was not observed in the resistance mesenteric or conduit renal arteries of age-matched SHRs. Furthermore, our experimental setup revealed two potential candidate proteins (Fbln5 and Cdh13), whose expressions were critically changed in cerebral arteries compared to systemic arteries at the early-onset of hypertension in the SHR.

In order to prevent neurovascular complications associated with hypertension, we need a better overview of the changes occurring in the cerebrovasculature at the initial stages of the disease. Our proteomic analysis identified 125 significantly regulated proteins that changed in the early-onset of hypertension in the SHR model. Our enrichment analysis of these proteins revealed that *Glyoxylate and dicarboxylate metabolism* was the most predominant pathway affected. Glyoxylate is the conjugant base of glyoxylic acid and involved in pH regulation. Five of the associated proteins are also directly (Cs, Aco2) or indirectly (Acss1, Pcca, Pccb) involved in the citric acid cycle and thus oxidation of carbohydrates and fatty acids. The protein expression of all five proteins was significantly upregulated in the cerebral arteries from the SHR compared to the WKY control at the early-onset of hypertension. A continued activation of the citric acid cycle together with upregulation of reactive oxygen species homeostasis was observed in resistant hypertensive patients (18). Excessive elevation of ROS can damage the vascular wall leading to vascular remodeling through a multitude of pathways (19). These protein and pathway changes occurred in 12-week-old SHR rats, supporting a critical mechanistic impact on cerebral arteries at the early-onset of hypertension.

Previously, we showed that ECM-related proteins were highly dysregulated in the resistance mesenteric arteries of the 12-week-old SHR. In this study, we repeated that analysis enabling us to compare the mesenteric and renal artery ECM changes in the SHR with the cerebral artery. In the cerebral artery, we detected 46 significantly regulated ECM-associated proteins compared to 97 and 66 proteins in mesenteric and renal arteries, respectively. The decreased number of ECM-related protein changes in the SHR cerebral arteries suggest that they are not remodeled to the same extent in early-onset hypertension as systemic arteries in the SHR, as has been observed previously (13, 20). Analysis of media-to-lumen ratio changes in the cerebral arteries of the WKY and SHR support these previous findings, showing that although the SHR cerebral arteries displayed some level of remodeling, the remodeling observed in the SHR mesenteric arteries was far greater (7). In line with the limited ECM remodeling in SHR cerebral arteries, we could not separate clearly the 12-week-old SHR and WKY control when using unsupervised hierarchical clustering or PCA based on the ECM-associated proteins (component 2–11%), whereas a clear separation was observed in mesenteric (component 1–31%) and renal arteries (component 2–22%).

Although structural arterial remodeling (inward eutrophic and hypertrophic) is an important factor known to contribute to increased total peripheral resistance in hypertension, vascular rarefication is another factor that can contribute to the increased resistance. Rarefaction can be due to fewer vessels connected in parallel or due to a total closure of some of the vessels (21). Vascular rarefaction is associated with the development of several clinical complications, such as cerebral microhemorrhages and lacunar infarcts (22). Reduced vascular density and angiogenesis has been demonstrated in the SHR previously (23). The balance of pro- and anti-angiogenesis proteins controls the dynamic ability of arterioles to grow, recede, or maintain their structure, thereby controlling vascular rarefaction (1). In this study, we have identified key angiogenic proteins that are dysregulated in the cerebral arteries in early-onset hypertension. By assigning the proteins as either pro- or anti-angiogenic, we show a clear pattern indicating that angiogenesis is likely to be impaired in the cerebral arteries of the SHR, which would promote vascular rarefication. A similar number of significantly regulated angiogenic proteins were identified in the SHR cerebral, mesenteric, and renal arteries. Using PCA plots, we detected a clear separation between SHR and WKY in cerebral arteries, a partial separation in mesenteric arteries, while no separation was observed in renal artery samples. Importantly, the pattern of increased anti-angiogenic proteins and decreased pro-angiogenic proteins was only observed in the SHR cerebral arteries and not mirrored in the SHR mesenteric and renal arteries. Additionally, this pattern was not observed in 6-week-old SHR cerebral arteries. Thus, early in the onset of hypertension, when systemic vessels undergo more prominent ECM-dependent inward remodeling in response to the increased transmural pressure, the cerebral arteries display a shift towards reduced angiogenesis, a precursor for rarefaction.

From the top 5 upregulated angiogenic proteins, three have previously been directly linked to hypertension. Scg2 (Secretogranin II) is a precursor of various neuropeptides, such as secretoneurin, and a genetic polymorphism in African-American subjects has been associated with hypertension (24). Mfge8 or Milk Fat Globule-EGF Factor 8 is a glycoprotein involved in several biological processes, including phenotypic modulation of vascular smooth muscle cells, apoptosis, and inflammation (25). We previously reported an upregulation of Mfge8 in mesenteric arteries from hypertensive rats (7). Neurofibromin 1 is a multifunctional protein involved in multiple signaling pathways. For example, Nf1 was reported to modulate vascular smooth muscle cell homeostasis in the vascular wall *via* the RAS signaling pathway (26). Genetic mutations in human *NF1* is associated with the disease *Neurofibromatosis type 1*, which is associated with an increased risk of hypertension (27).

From the top 5 downregulated angiogenic proteins, four have been linked to hypertension previously. Anxa8, or Annexin A8, is a calcium-dependent phospholipid-binding protein that is involved in membrane trafficking, ion channel activity, and anti-inflammation (28). Anxa8 was identified to play an important role for the induction of hypertension in salt-sensitive rats (29). Serpina6, also known as corticosteroid-binding globulin, is a carrier protein that binds and transports cortisol and other glucocorticoid hormones (30). A genetic variant of Serpina6 causing reduced cortisol-binding affinity has been associated with hypertension (31). Ctss, or cathepsin S, is a lysosomal cysteine protease involved in antigen presentation, ECM remodeling. Ctss is a potent protease cleaving elastin in the arterial wall (32), and upregulation of CTSS has been observed in lung tissue from human patients with pulmonary arterial hypertension (33). Interestingly, Ctss was upregulated in our renal artery dataset (logFC = 0.14) but downregulated in mesenteric and cerebral arteries (logFC = −0.30 and −0.59, respectively), suggesting an altered expression depending on the arterial bed. Lgals3, or galectin-3, is a carbohydrate-binding protein involved in several cellular processes, such as cellular growth, proliferation, and cell–matrix interactions (34). Increased galectin-3 serum levels have been observed in hypertensive patients with left ventricular remodeling (35). The information about these highly regulated proteins in relation to cerebral artery function in hypertension is currently limited. The changes demonstrate a widespread pathological impact of high blood pressure and thus provide novel insight into the underlying pathological mechanisms.

Fbln5 (Fibulin 5 or DANCE) was identified as a candidate protein that was expressed differentially in the cerebral arteries compared to two systemic vascular beds at the early-onset of hypertension in the SHR. Fbln5 is involved in the arrangement of elastic fibers in the vessel wall and has been reported to suppress the matrix metallopeptidase MMP9 (36). Mice that lack Fbln5 display fragmented and disorganized elastic fibers (37). In contrast, overexpression of Fbln5 was found to increase elastin deposition in retinal pigment epithelial cells (38). Based on the morphological and proteomic differences that were observed between these vascular beds, future work should investigate whether Fbln5 is a critical mediator of the vascular bed–associated difference. Interestingly, a phenome-wide association analysis of *FBLN5* in the white British participants of the UK Biobank identified an intronic single nucleotide polymorphism (rs1049468267) within the *FBLN5* gene that was associated with “cerebral atherosclerosis” (*p* = 9.9 × 10-7; effect size [beta value] = 88) and “occlusion and stenosis of precerebral arteries” (*p* = 2.0 × 10-4; effect size [beta value] = 16). These data indicate a strong association of Fbln5 with cerebral artery diseases, and our data suggest that this protein is dysregulated in cerebral arteries early in hypertension, which may increase the risk of certain cerebrovascular diseases related to hypertension.

The second candidate protein identified was Cdh13 (also Cadherin 13 or T-cadherin). Cdh13 can work as receptor for low-density lipoproteins and adiponectin and has demonstrated potential navigating functions in migrating cells (39). Its location was found to be restricted to caveolae in aortic smooth muscle cells where it interacted with Src, supporting that Cdh13 can facilitate intracellular signaling (39). Genome-wide association studies have identified genetic variants in *CDH13* that are associated with coronary artery disease (40), blood pressure traits (41), and hypertension (42). Furthermore, increased Cdh13 expression was found to promote tumor angiogenesis *in vivo* (43). Taken together, the changes in expression of these candidate proteins have direct impacts on vascular remodeling and angiogenesis. However, the molecular pathways driving these changes and whether they are connected should be investigated in future studies. Importantly, the regulation of both candidate proteins are early mechanistic changes in the cerebrovasculature induced by elevated blood pressure. With further investigation, these proteins have the potential to be innovative therapeutic targets reducing the long-term risk of developing brain diseases associated with hypertension.

This study has some limitations; first, because the cerebral arteries were rapidly dissected to prevent protein degradation, leftover material from the meninges cannot be excluded. Second, the study was limited to the circle of Willis and the primary cerebral arteries, meaning that the arteries have been mainly conduit arteries with lack of brain-penetrating arteries. However, by using these arteries, we diminished the impact of our first limitation by reducing the inclusion of proteins derived from brain cells, for example, neurons, glial cells, and pericytes. Third, the study did not investigate the presence of rarefaction or blood flow changes because of the early phase of the disease. We consider that such changes would not be present at this time point but rather develop over time. Future studies should therefore investigate these parameters in older SHRs that may yield a different picture of the vascular response to elevated blood pressure (14).

In summary, this study has discovered critical proteomic differences in the cerebrovasculature of hypertensive rats. In-depth protein profiling has identified a hypertension-induced imbalance of angiogenic proteins in cerebral arteries that upregulate anti-angiogenic proteins and downregulate pro-angiogenic proteins. Collectively, these data reveal novel protein changes in the cerebrovasculature at the early-onset of hypertension that, over-time, could promote to cerebrovascular rarefaction, thereby increasing the risk of cerebrovascular diseases associated with hypertension. By mapping the hypertension-associated changes in the cerebral artery wall, this study reveals potential novel proteins and pathways that could be targeted to reduce the risk of developing cerebrovascular diseases associated with hypertension.

## Experimental procedures

### Experimental animals

The animal experiments were approved by local Animal Care and Use Committees. Experiments were performed in accordance with the directives of the Danish National Committee on Animal Research Ethics and Danish legislation on experimental animals. Rats were made unconscious by a single percussive blow to the head, in accordance with the methods of killing animals described in annex IV of the EU Directive 2010/63EU. Directly after the onset of unconsciousness, cervical dislocation was used to euthanize the rats. Cohorts of male SHRs (SHR/KyoRj) and WKYs (WKY/KyoRj) at 6 and 12 weeks of age were group housed and supplied with *ad libitum* water and food access. Rats were kept on a 12 h/12 h light/dark cycle and were transferred to clean cages regularly.

### Dissection of cerebral arteries

Following cervical dislocation, the brain was excised delicately and placed in cold physiological salt solution (121 mM NaCl; 2.8 mM KCl; 1.2 mM KH_2_PO_4_; 1.2 mM MgSO_4_; 25 mM NaHCO_3_; 1.6 mM CaCl_2_; 0.03 mM EDTA; 5.5 mM D-glucose) saturated with carbogen (O_2_ 95%; CO_2_ 5%) at pH 7.4. The circle of Willis with primary cerebral arteries (posterior, anterior, and middle cerebral arteries) were collected in 1.5 ml Lobind centrifugation tubes (Eppendorf), snap frozen in liquid nitrogen, and stored at −80 °C.

A small segment (2–3 mm) of basilar artery from a subgroup of rats was embedded in Tissue-Tek OCT (Sakura) for sectioning and staining.

### Staining and microscopy imaging

Arterial cross sections were collected using a cryostat microtome (Leica CM3050 S). Sections were cut at 12 μm thickness and attached to Superfrost Plus glass slides (VWR) and stored at −80 °C. Arterial sections were stained using Sirius red for connective tissue, as reported previously (7). Slides were scanned using a ZEISS Axioscan 7 slide scanner with 20×/0.8 Plan-Apochromat objective lens (Zeiss). Images were cropped to individual arterial cross sections and analyzed, blinded, in ZEN (v3.2, blue edition, Zeiss) software. Media and lumen diameters were measured using a profile ruler tool to calculate the media-to-lumen ratio.

For immunohistochemistry, whole brain tissue were fixed in 4% PFA/1×PBS overnight at 4 °C followed by washing in 1×PBS. Tissue sections were cut at 16 μm thickness and attached to Superfrost Plus glass slides (VWR). Tissue sections were blocked in blocking buffer (5% normal swine serum (Jackson ImmunoResearch), 1% bovine serum albumin (BSA, Sigma), 0.1% TritonX-100 (Sigma) in 1 × PBS) and stained with commercial anti-alpha smooth muscle actin antibody [1A4] (ab7817, 1:400, Abcam), anti-Fibulin 5 antibody (ab202977, 1:200, Abcam), and anti-human cadherin-13 antibody (AF3264, 1:200, R&D Systems) diluted in 1% BSA, 0.1% TritonX-100 in 1×PBS overnight at 4 °C. Sections were counterstained with commercial anti-mouse, anti-rabbit, and anti-goat secondary antibodies cross-absorbed to Alexa Flour 488, 564, and 647, respectively, (1:400, Invitrogen) diluted in 1% BSA, 0.1% TritonX-100 in 1×PBS for 1 h at RT. Washes in washing buffer (0.25% BSA, 0.1% TritonX-100 in 1×PBS) were used between and after antibody staining. Hoechst 33342 (1:1000 in 1×PBS, Invitrogen) was used after the secondary antibody staining. Sections were mounted in anti-fade mounting medium (SlowFade Diamond Antifade Mountant, S36967, Invitrogen).

Fluorescent images were acquired on a LSM900 confocal imaging setup (Zeiss) using a 20×/0.8 Plan-Apochromat Air objective lens (Zeiss). The intensity was measured using a profile ruler tool in ZEN (3.2, blue edition, Zeiss) to acquire the max intensity across a line spanning over the vessel wall. Statistical analysis was performed in GraphPad Prism (v9) using unpaired Student’s *t* test.

### Protein isolation and quantification

The cerebral arteries were homogenized in 100 μl of ice-cold lysis buffer (50 mM Tris pH 8.5, 5 mM EDTA pH 8.0, 150 mM NaCl, 10 mM KCl, 1% NP-40, and 1×complete protease inhibitor cocktail (Roche)) by three rounds of chopping the tissue using dissection scissors and a handheld homogenizer (7). Medial PFC regions (0.3 cm × 0.3 cm × 0.3 cm) were homogenized in 150 μl lysis buffer. Homogenates were centrifuged at 11, 000*g* for 10 min at 4 °C to obtain the supernatant. Protein quantification of the tissue extracts was determined by bicinchoninic acid assay (Thermo Fisher Scientific).

### Sample preparation for mass spectrometry analysis

Digestion buffer containing 0.5% sodium deoxycholate in 50 mM triethylammonium bicarbonate was added to the homogenized tissue extracts (100 μg protein) followed by heat-treatment for 5 min at 95 °C. Samples were cooled on ice and prepared by filter-aided sample preparation (44) using flat spin filters (Microcon-10 kDa). Samples were reduced and alkylated in digestion buffer containing 1:50 (v:v) tris(2-carboxyethyl)phosphine (0.5 M, Sigma) and 1:10 (v:v) 2-chloroacetamide (0.5 M, Sigma) for 30 min at 37 °C. Samples were digested overnight at 37 °C with 1 μg Trypsin/LysC mix (Promega) and 0.01% ProteaseMAX (Promega). Centrifugation at 14, 000*g* for 15 min was used in between the different steps. Peptides were desalted using stage-tips containing a Poly-styrene-divinylbenzene copolymer modified with sulfonic acid groups (SDB-RPS; 3M) material, as described previously (7). In brief, samples were mixed with 5× volume 99% isopropanol (Sigma)/1% TFA (Sigma), loaded to the stage-tips, and centrifuged at 1, 500*g* at 4 °C until passed through the SDB-RPS filter. Each filter was washed twice in 99% isopropanol (Sigma)/1% TFA (Sigma) and 0.2% TFA, respectively. Samples were eluted using 80% acetonitrile (Sigma)/2% Ammonia (25%, Sigma). After vacuum centrifugation, samples were resuspended in 2% acetonitrile (Sigma)/0.1% TFA (Sigma).

### Data acquisition by DIA-based mass spectrometry

Samples were analyzed on a Bruker timsTOF Pro mass spectrometer (Bruker Daltonics) in the positive ion mode with a Captivespray ion source on-line connected to a Dionex Ultimate 3000RSnano chromatography systems (Thermo Fisher Scientific). Peptides were separated on an Aurora column with captive spray insert (C18 1.6 uM particles, 25 cm, 75 μm inner diameter; IonOptics) at 60 °C. A solvent gradient of buffer A (0.1 % formic acid) and buffer B (99.9% acetonitrile/0.1% formic acid) over 140 min, at a flow rate of 600 nl/min, were used, respectively. The mass spectrometer was operated in DIA PASEF mode with 1.1 s cycle time and TIMS ramp time of 100.0 ms. MS scan range was set to 100 to 1700 m/z.

### Protein identification by mass spectrometry

Raw DIA files were analyzed with DIA-NN software (v.1.8.0) (https://github.com/vdemichev/diann) (45) using UniProt FASTA database (UP000002494_10116.fasta (21,587 entries) and UP000002494_10116_additional.fasta (9981 entries), August 2020) and deep learning–based spectra to generate a library. In DIA-NN, the options ‘FASTA digest for library-free search/library generation’ and ‘Deep learning-based spectra, RTs, and IMs prediction’ were enabled and all other settings were left default: Digestion protease = Trypsin/P; missed cleavage = 1; max number of variable modifications = 0; N-term M excision = True; C carbamidomethylation = True; peptide length range = 7 to 30; precursor charge range = 1 to 4; precursor m/z range = 300 to 1800; fragment ion m/z range = 200 to 1800; Precursor false discovery rate (FDR) (%) = 1%; Use isotopologs = enabled; MBR = enabled; Neural network classifier = single-pass mode; Protein inference = Genes; Quantification strategy = Any LC (high accuracy); Cross-run normalization = RT-dependent; Library generation = Smart profiling; Speed and RAM usage = Optimal results.

MS raw files from a previous publication (7) were downloaded from the ProteomeXchange Consortium *via* PRIDE with the identifier PXD026051. The files contained DIA-MS information about mesenteric and renal arteries from 12-week-old SHR and WKY. The DIA-NN software was used for protein identification with the same settings as listed above.

### Bioinformatic analysis

Number of unique proteins at 1% FDR were obtained from the report file generated by DIA-NN (45). Protein quantities were obtained from the unique gene list in DIA-NN (*report.unique_genes_matrix.tsv*) and implemented in Perseus (v1.6.14.0) (46). Data was log2 transformed and filtered for by three valid values in at least one group. Statistical comparisons between 12-week-old rat groups was made using the limma package for R (15, 47) to enable batch correction and calculation of statistical significance levels and fold change difference per protein. Furthermore, two-sided Student’s *t* test in Perseus was used to compare PFC groups, pre-hypertensive groups, as well as previously published mesenteric and renal artery data (7), with permutation-based FDR (<0.05) and 250 randomizations. Missing values were only imputed for PCA (width =0.4, down shift = 1.8). ECM and angiogenesis enrichments were achieved by comparing with curated gene lists(16, 17, 48, 49) and selecting overlapping proteins for further analysis. The pro- or anti-angiogenic property of angiogenesis-associated proteins was manually determined by literature mining (Tables S1-4). Hierarchical clustering was created based on z-scored LFQ values and generated by average linkage, preprocessing with k-means, and Euclidean distance. The z-score normalization was calculated by subtracting mean intensity from each protein value across all samples followed by division by the standard deviation (7). ClueGO (v2.5.8) (50) in Cytoscape (v3.9.1) (51) was used to generate functionally grouped networks and enrichment analysis of significantly regulated proteins. The enrichment analysis was performed as previously described (7); *Rattus norvegicus* was selected as organism, and the GO biological processes (GO-BiologicalProcess, CellularComponent, ImmuneSystemProcess, MolecularFunction-EBI-UniProt-GOA-ACAP-ARAP, downloaded 15.01.2021) and KEGG (downloaded 15.01.2021) were used as ontology reference sets. The following settings were used; GO tree interval = 3 to 8; GO term/pathway selection = 3 min #Genes, 4% genes; Kappa-score = 0.4; enrichment/depletion (two-sided hypergeometric test with Bonferroni step down, *p* ≤ 0.05); GO term fusion was enabled.

## Data availability

The mass spectrometry proteomics data have been deposited to the ProteomeXchange Consortium *via* the PRIDE (52) partner repository with the dataset identifier PXD040818.

## Supporting information

This article contains supporting information.

## Conflict of interest

The authors declare that they have no conflicts of interest with the contents of this article.

## Supporting information

Table S4

Supplementary References
