# Supplementary material for: Proteomic mapping reveals dysregulated angiogenesis in the cerebral arteries of rats with early-onset hypertension
Source: J Biol Chem. 2023 Sep 1;299(10):105221. doi: 10.1016/j.jbc.2023.105221 (PMC10558802; doi:10.1016/j.jbc.2023.105221)
Supplement: Supplementary References [file mmc5.docx]

**Supplementary references in Tables S1-4:**

| 1 | doi: 10.1161/CIRCRESAHA.113.301686 | Adam, M. G., Berger, C., Feldner, A., Yang, W.-J., Wüstehube-Lausch, J., Herberich, S. E., Pinder, M., Gesierich, S., Hammes, H.-P., Augustin, H. G., and Fischer, A. (2013) Synaptojanin-2 Binding Protein Stabilizes the Notch Ligands DLL1 and DLL4 and Inhibits Sprouting Angiogenesis. Circ. Res. 113, 1206–1218 |
| --- | --- | --- |
| 2 | doi: 10.1016/j.molimm.2020.02.020 | Babaie, F., Hosseinzadeh, R., Ebrazeh, M., Seyfizadeh, N., Aslani, S., Salimi, S., Hemmatzadeh, M., Azizi, G., Jadidi-Niaragh, F., and Mohammadi, H. (2020) The roles of ERAP1 and ERAP2 in autoimmunity and cancer immunity: New insights and perspective. Mol. Immunol. 121, 7–19 |
| 3 | doi: 10.1158/0008-5472.CAN-05-3612 | Battle, T. E., Lynch, R. A., and Frank, D. A. (2006) Signal Transducer and Activator of Transcription 1 Activation in Endothelial Cells Is a Negative Regulator of Angiogenesis. *Cancer Res.* **66**, 3649–3657 |
| 4 | doi: 10.1096/fj.10-180604 | Boutté, A. M., Friedman, D. B., Bogyo, M., Min, Y., Yang, L., and Lin, P. C. (2011) Identification of a myeloid‐derived suppressor cell cystatin‐like protein that inhibits metastasis. *FASEB J.* **25**, 2626–2637 |
| 5 | doi: 10.1074/jbc.M507401200 | Cai, J., Jiang, W. G., Grant, M. B., and Boulton, M. (2006) Pigment Epithelium-derived Factor Inhibits Angiogenesis via Regulated Intracellular Proteolysis of Vascular Endothelial Growth Factor Receptor 1. *J. Biol. Chem.* **281**, 3604–3613 |
| 6 | doi: 10.1158/0008-5472.CAN-05-0848 | Chigurupati, S., Kulkarni, T., Thomas, S., and Shah, G. (2005) Calcitonin Stimulates Multiple Stages of Angiogenesis by Directly Acting on Endothelial Cells. *Cancer Res.* **65**, 8519–8529 |
| 7 | doi: 10.1007/s11906-003-0072-3 | Corvol, P., Lamandé, N., Cruz, A., Celerier, J., and Gasc, J.-M. (2003) Inhibition of angiogenesis: A new function for angiotensinogen and des(angiotensin I)angiotensinogen. *Curr. Hypertens. Rep.* **5**, 149–154 |
| 8 | doi: 10.1371/journal.pone.0002518 | Destouches, D., El Khoury, D., Hamma-Kourbali, Y., Krust, B., Albanese, P., Katsoris, P., Guichard, G., Briand, J. P., Courty, J., and Hovanessian, A. G. (2008) Suppression of Tumor Growth and Angiogenesis by a Specific Antagonist of the Cell-Surface Expressed Nucleolin. PLoS One. 3, e2518 |
| 9 | doi: 10.1007/s10456-008-9101-9 | ten Dijke, P., Goumans, M.-J., and Pardali, E. (2008) Endoglin in angiogenesis and vascular diseases. *Angiogenesis*. **11**, 79–89 |
| 10 | doi: 10.3389/fendo.2022.906586 | Duval, V., Alayrac, P., Silvestre, J.-S., and Levoye, A. (2022) Emerging Roles of the Atypical Chemokine Receptor 3 (ACKR3) in Cardiovascular Diseases. *Front. Endocrinol. (Lausanne).* **13**, 1–11 |
| 11 | doi: 10.1038/s41586-018-0466-7 | Eelen, G., Dubois, C., Cantelmo, A. R., Goveia, J., Brüning, U., DeRan, M., Jarugumilli, G., van Rijssel, J., Saladino, G., Comitani, F., Zecchin, A., Rocha, S., Chen, R., Huang, H., Vandekeere, S., Kalucka, J., Lange, C., Morales-Rodriguez, F., Cruys, B., Treps, L., Ramer, L., Vinckier, S., Brepoels, K., Wyns, S., Souffreau, J., Schoonjans, L., Lamers, W. H., Wu, Y., Haustraete, J., Hofkens, J., Liekens, S., Cubbon, R., Ghesquière, B., Dewerchin, M., Gervasio, F. L., Li, X., van Buul, J. D., Wu, X., and Carmeliet, P. (2018) Role of glutamine synthetase in angiogenesis beyond glutamine synthesis. Nature. 561, 63–69 |
| 12 | doi: 10.1002/1878-0261.13044 | Fang, C., Dai, L., Wang, C., Fan, C., Yu, Y., Yang, L., Deng, H., and Zhou, Z. (2021) Secretogranin II impairs tumor growth and angiogenesis by promoting degradation of hypoxia‐inducible factor‐1α in colorectal cancer. *Mol. Oncol.* **15**, 3513–3526 |
| 13 | doi: 10.1038/onc.2011.146 | Gerstel, D., Wegwitz, F., Jannasch, K., Ludewig, P., Scheike, K., Alves, F., Beauchemin, N., Deppert, W., Wagener, C., and Horst, A. K. (2011) CEACAM1 creates a pro-angiogenic tumor microenvironment that supports tumor vessel maturation. Oncogene. 30, 4275–4288 |
| 14 | doi: 10.1038/s41598-019-50866-x | Gómez-Escudero, J., Clemente, C., García-Weber, D., Acín-Pérez, R., Millán, J., Enríquez, J. A., Bentley, K., Carmeliet, P., and Arroyo, A. G. (2019) PKM2 regulates endothelial cell junction dynamics and angiogenesis via ATP production. *Sci. Rep.* **9**, 15022 |
| 15 | doi: 10.1158/0008-5472.CAN-07-2953 | Hebbard, L. W., Garlatti, M., Young, L. J. T., Cardiff, R. D., Oshima, R. G., and Ranscht, B. (2008) T-cadherin Supports Angiogenesis and Adiponectin Association with the Vasculature in a Mouse Mammary Tumor Model. *Cancer Res.* **68**, 1407–1416 |
| 16 | doi: 10.1210/jc.2003-030243 | Herr, F., Liang, O. D., Herrero, J., Lang, U., Preissner, K. T., Han, V. K. M., and Zygmunt, M. (2003) Possible Angiogenic Roles of Insulin-Like Growth Factor II and Its Receptors in Uterine Vascular Adaptation to Pregnancy. J. Clin. Endocrinol. Metab. 88, 4811–4817 |
| 17 | doi: 10.1242/jcs.091066 | Hetheridge, C., Scott, A. N., Swain, R. K., Copeland, J. W., Higgs, H. N., Bicknell, R., and Mellor, H. (2012) The novel formin FMNL3 is a cytoskeletal regulator of angiogenesis. *J. Cell Sci.* **125**, 1420–1428 |
| 18 | doi: 10.1186/2045-824X-4-1 | Howe, G. A., and Addison, C. L. (2012) RhoB controls endothelial cell morphogenesis in part via negative regulation of RhoA. *Vasc. Cell*. **4**, 1 |
| 19 | doi.org/10.1242/dev.02538 | Ishimura, A., Ng, J. K., Taira, M., Young, S. G., and Osada, S.-I. (2006) Man1, an inner nuclear membrane protein, regulates vascular remodeling by modulating transforming growth factor β signaling. *Development*. **133**, 3919–3928 |
| 20 | doi: 10.1161/01.CIR.0000158487.80483.09 | Jacobi, J., Sydow, K., von Degenfeld, G., Zhang, Y., Dayoub, H., Wang, B., Patterson, A. J., Kimoto, M., Blau, H. M., and Cooke, J. P. (2005) Overexpression of Dimethylarginine Dimethylaminohydrolase Reduces Tissue Asymmetric Dimethylarginine Levels and Enhances Angiogenesis. Circulation. 111, 1431–1438 |
| 21 | doi.org/10.1186/s12864-020-6456-9 | Jahejo, A. R., Zhang, D., Niu, S., Mangi, R. A., Khan, A., Qadir, M. F., Khan, A., Chen, H., and Tian, W. (2020) Transcriptome-based screening of intracellular pathways and angiogenesis related genes at different stages of thiram induced tibial lesions in broiler chickens. BMC Genomics. 21, 50 |
| 22 | doi: 10.1016/j.matbio.2015.01.023 | Järveläinen, H., Sainio, A., and Wight, T. N. (2015) Pivotal role for decorin in angiogenesis. *Matrix Biol.* **43**, 15–26 |
| 23 | doi:10.1158/1078-0432.CCR-14-1236 | Karikoski, M., Marttila-Ichihara, F., Elima, K., Rantakari, P., Hollmén, M., Kelkka, T., Gerke, H., Huovinen, V., Irjala, H., Holmdahl, R., Salmi, M., and Jalkanen, S. (2014) Clever-1/Stabilin-1 Controls Cancer Growth and Metastasis. Clin. Cancer Res. 20, 6452–6464 |
| 24 | doi: 10.1091/mbc.e07-12-1215 | Katsuno, T., Umeda, K., Matsui, T., Hata, M., Tamura, A., Itoh, M., Takeuchi, K., Fujimori, T., Nabeshima, Y., Noda, T., Tsukita, S., and Tsukita, S. (2008) Deficiency of Zonula Occludens-1 Causes Embryonic Lethal Phenotype Associated with Defected Yolk Sac Angiogenesis and Apoptosis of Embryonic Cells. Mol. Biol. Cell. 19, 2465–2475 |
| 25 | doi: 10.1172/JCI66171 | Klauber-DeMore, N. (2012) Are epsins a therapeutic target for tumor angiogenesis? *J. Clin. Invest.* **122**, 4341–4343 |
| 26 | doi: 10.1161/CIRCRESAHA.119.315023 | Korf-Klingebiel, M., Reboll, M. R., Grote, K., Schleiner, H., Wang, Y., Wu, X., Klede, S., Mikhed, Y., Nobre, A., Bauersachs, J., Klintschar, M., Rudat, C., Kispert, A., Niessen, H. W., Lübke, T., Dierks, T., and Wollert, K. C. (2019) Heparan Sulfate–Editing Extracellular Sulfatases Enhance VEGF Bioavailability for Ischemic Heart Repair. Circ. Res. 125, 787–801 |
| 27 | doi: 10.1016/j.bbcan.2003.09.004 | Kranenburg, O., Gebbink, M. F. B. G., and Voest, E. E. (2004) Stimulation of angiogenesis by Ras proteins. *Biochim. Biophys. Acta - Rev. Cancer*. **1654**, 23–37 |
| 28 | doi: 10.1016/j.bbrc.2010.10.043 | Kubota, R., Numaguchi, Y., Ishii, M., Niwa, M., Okumura, K., Naruse, K., and Murohara, T. (2010) Ischemia-induced angiogenesis is impaired in aminopeptidase A deficient mice via down-regulation of HIF-1α. *Biochem. Biophys. Res. Commun.* **402**, 396–401 |
| 29 | doi: 10.1093/hmg/dds346 | Kuo, D. S., Labelle-Dumais, C., and Gould, D. B. (2012) COL4A1 and COL4A2 mutations and disease: insights into pathogenic mechanisms and potential therapeutic targets. *Hum. Mol. Genet.* **21**, R97–R110 |
| 30 | doi:10.1016/j.yjmcc.2017.07.001 | Kurusamy, S., López-Maderuelo, D., Little, R., Cadagan, D., Savage, A. M., Ihugba, J. C., Baggott, R. R., Rowther, F. B., Martínez-Martínez, S., Arco, P. G., Murcott, C., Wang, W., Francisco Nistal, J., Oceandy, D., Neyses, L., Wilkinson, R. N., Cartwright, E. J., Redondo, J. M., and Armesilla, A. L. (2017) Selective inhibition of plasma membrane calcium ATPase 4 improves angiogenesis and vascular reperfusion. J. Mol. Cell. Cardiol. 109, 38–47 |
| 31 | doi: 10.1158/0008-5472.CAN-04-4012 | Lamagna, C., Hodivala-Dilke, K. M., Imhof, B. A., and Aurrand-Lions, M. (2005) Antibody against Junctional Adhesion Molecule-C Inhibits Angiogenesis and Tumor Growth. *Cancer Res.* **65**, 5703–5710 |
| 32 | doi: 10.1042/BST20140244 | Lampropoulou, A., and Ruhrberg, C. (2014) Neuropilin regulation of angiogenesis. *Biochem. Soc. Trans.* **42**, 1623–1628 |
| 33 | doi: 10.1161/01.res.82.8.845 | Lee, W.-S., Jain, M. K., Arkonac, B. M., Zhang, D., Shaw, S.-Y., Kashiki, S., Maemura, K., Lee, S.-L., Hollenberg, N. K., Lee, M.-E., and Haber, E. (1998) Thy-1, a Novel Marker for Angiogenesis Upregulated by Inflammatory Cytokines. *Circ. Res.* **82**, 845–851 |
| 34 | doi: 10.1161/ATVBAHA.116.308422 | Lee, H.-W., Chong, D. C., Ola, R., Dunworth, W. P., Meadows, S., Ka, J., Kaartinen, V. M., Qyang, Y., Cleaver, O., Bautch, V. L., Eichmann, A., and Jin, S.-W. (2017) Alk2/ACVR1 and Alk3/BMPR1A Provide Essential Function for Bone Morphogenetic Protein–Induced Retinal Angiogenesis. Arterioscler. Thromb. Vasc. Biol. 37, 657–663 |
| 35 | doi: 10.1096/fj.201902899R | Lee, S., Kim, H., Naidansuren, P., Ham, K. A., Choi, H. S., Ahn, H., Kim, M., Kang, D. H., Kang, S. W., and Joe, Y. A. (2020) Peroxidasin is essential for endothelial cell survival and growth signaling by sulfilimine crosslink‐dependent matrix assembly. FASEB J. 34, 10228–10241 |
| 36 | doi: 10.1016/j.molonc.2013.10.003 | Leelahavanichkul, K., Amornphimoltham, P., Molinolo, A. A., Basile, J. R., Koontongkaew, S., and Gutkind, J. S. (2014) A role for p38 MAPK in head and neck cancer cell growth and tumor-induced angiogenesis and lymphangiogenesis. *Mol. Oncol.* **8**, 105–118 |
| 37 | doi: 10.1097/SLA.0000000000003522 | Leick, K. M., Rodriguez, A. B., Melssen, M. M., Benamar, M., Lindsay, R. S., Eki, R., Du, K.-P., Parlak, M., Abbas, T., Engelhard, V. H., and Slingluff, C. L. (2019) The Barrier Molecules Junction Plakoglobin, Filaggrin, and Dystonin Play Roles in Melanoma Growth and Angiogenesis. Ann. Surg. 270, 712–722 |
| 38 | doi: 10.1074/jbc.M302028200 | Lin, C. G., Leu, S.-J., Chen, N., Tebeau, C. M., Lin, S.-X., Yeung, C.-Y., and Lau, L. F. (2003) CCN3 (NOV) Is a Novel Angiogenic Regulator of the CCN Protein Family. *J. Biol. Chem.* **278**, 24200–24208 |
| 39 | doi: 10.1161/CIRCRESAHA.110.218271 | Liu, H., Zhang, W., Kennard, S., Caldwell, R. B., and Lilly, B. (2010) Notch3 Is Critical for Proper Angiogenesis and Mural Cell Investment. *Circ. Res.* **107**, 860–870 |
| 40 | doi: 10.7150/jca.19452 | Liu, J., and Hoh, J. (2017) Loss of Complement Factor H in Plasma Increases Endothelial Cell Migration. *J. Cancer*. **8**, 2184–2190 |
| 41 | doi: 10.7150/thno.30305 | Liu, J., Wada, Y., Katsura, M., Tozawa, H., Erwin, N., Kapron, C. M., Bao, G., and Liu, J. (2018) Rho-Associated Coiled-Coil Kinase (ROCK) in Molecular Regulation of Angiogenesis. *Theranostics*. **8**, 6053–6069 |
| 42 | doi: 10.1038/onc.2011.487 | Lorenzon, E., Colladel, R., Andreuzzi, E., Marastoni, S., Todaro, F., Schiappacassi, M., Ligresti, G., Colombatti, A., and Mongiat, M. (2012) MULTIMERIN2 impairs tumor angiogenesis and growth by interfering with VEGF-A/VEGFR2 pathway. Oncogene. 31, 3136–3147 |
| 43 | doi: 10.1016/j.cellsig.2019.06.007 | MacKeil, J. L., Brzezinska, P., Burke-Kleinman, J., Theilmann, A. L., Nicol, C. J. B., Ormiston, M. L., and Maurice, D. H. (2019) Phosphodiesterase 3B (PDE3B) antagonizes the anti-angiogenic actions of PKA in human and murine endothelial cells. Cell. Signal. 62, 109342 |
| 44 | doi.org/10.3389/fnins.2018.00821 | Miller, D. J., and Fort, P. E. (2018) Heat Shock Proteins Regulatory Role in Neurodevelopment. *Front. Neurosci.* **12**, 1–15 |
| 45 | doi: 10.1093/cvr/cvu260 | Moon, E.-H., Kim, Y. S., Seo, J., Lee, S., Lee, Y. J., and Oh, S. P. (2015) Essential role for TMEM100 in vascular integrity but limited contributions to the pathogenesis of hereditary haemorrhagic telangiectasia. *Cardiovasc. Res.* **105**, 353–360 |
| 46 | doi: 10.1161/ATVBAHA.111.232587 | Motegi, S., Leitner, W. W., Lu, M., Tada, Y., Sárdy, M., Wu, C., Chavakis, T., and Udey, M. C. (2011) Pericyte-Derived MFG-E8 Regulates Pathologic Angiogenesis. *Arterioscler. Thromb. Vasc. Biol.* **31**, 2024–2034 |
| 47 | doi:10.1111/micc.12372 | Nguyen, D. T., Gao, L., Wong, A., and Chen, C. S. (2017) Cdc42 regulates branching in angiogenic sprouting in vitro. *Microcirculation*. **24**, 139–148 |
| 48 | doi: 10.1046/j.1538-7836.2003.00182.x | Oh, C.-W., Hoover-Plow, J., and Plow, E. F. (2003) The role of plasminogen in angiogenesis in vivo. *J. Thromb. Haemost.* **1**, 1683–1687 |
| 49 | PMID: 29332977 | Olsen, J. J., Pohl, S. Ö.-G., Deshmukh, A., Visweswaran, M., Ward, N. C., Arfuso, F., Agostino, M., and Dharmarajan, A. (2017) The Role of Wnt Signalling in Angiogenesis. *Clin. Biochem. Rev.* **38**, 131–142 |
| 50 | doi: 10.1016/s0092-8674(00)81848-6 | O’Reilly, M. S., Boehm, T., Shing, Y., Fukai, N., Vasios, G., Lane, W. S., Flynn, E., Birkhead, J. R., Olsen, B. R., and Folkman, J. (1997) Endostatin: An Endogenous Inhibitor of Angiogenesis and Tumor Growth. *Cell*. **88**, 277–285 |
| 51 | doi: 10.1152/ajpcell.00139.2013 | Palenski, T. L., Gurel, Z., Sorenson, C. M., Hankenson, K. D., and Sheibani, N. (2013) Cyp1B1 expression promotes angiogenesis by suppressing NF-κB activity. *Am. J. Physiol. Physiol.* **305**, C1170–C1184 |
| 52 | doi: 10.1038/s41419-020-03304-0 | Pan, S., Zhao, X., Shao, C., Fu, B., Huang, Y., Zhang, N., Dou, X., Zhang, Z., Qiu, Y., Wang, R., Jin, M., and Kong, D. (2021) STIM1 promotes angiogenesis by reducing exosomal miR-145 in breast cancer MDA-MB-231 cells. *Cell Death Dis.* **12**, 38 |
| 53 | doi: 10.1016/j.bbrc.2005.10.004 | Park, J. E., Lee, D. H., Lee, J. A., Park, S. G., Kim, N.-S., Park, B. C., and Cho, S. (2005) Annexin A3 is a potential angiogenic mediator. *Biochem. Biophys. Res. Commun.* **337**, 1283–1287 |
| 54 | doi: 10.1016/j.jaut.2010.06.013 | Passam, F. H., Qi, J. C., Tanaka, K., Matthaei, K. I., and Krilis, S. A. (2010) In vivo modulation of angiogenesis by beta 2 glycoprotein I. *J. Autoimmun.* **35**, 232–240 |
| 55 | doi: 10.1016/j.ajpath.2014.07.012 | Patel, C., Narayanan, S. P., Zhang, W., Xu, Z., Sukumari-Ramesh, S., Dhandapani, K. M., Caldwell, R. W., and Caldwell, R. B. (2014) Activation of the Endothelin System Mediates Pathological Angiogenesis during Ischemic Retinopathy. Am. J. Pathol. 184, 3040–3051 |
| 56 | doi: 10.1038/s41388-017-0107-x | Paulitti, A., Andreuzzi, E., Bizzotto, D., Pellicani, R., Tarticchio, G., Marastoni, S., Pastrello, C., Jurisica, I., Ligresti, G., Bucciotti, F., Doliana, R., Colladel, R., Braghetta, P., Poletto, E., Di Silvestre, A., Bressan, G., Colombatti, A., Bonaldo, P., and Mongiat, M. (2018) The ablation of the matricellular protein EMILIN2 causes defective vascularization due to impaired EGFR-dependent IL-8 production affecting tumor growth. Oncogene. 37, 3399–3414 |
| 57 | doi: 10.1097/MOH.0b013e3282fdc69e | Perez-Pinera, P., Berenson, J. R., and Deuel, T. F. (2008) Pleiotrophin, a multifunctional angiogenic factor: mechanisms and pathways in normal and pathological angiogenesis. *Curr. Opin. Hematol.* **15**, 210–214 |
| 58 | doi: 10.1096/fj.11-200154 | Pi, L., Shenoy, A. K., Liu, J., Kim, S., Nelson, N., Xia, H., Hauswirth, W. W., Petersen, B. E., Schultz, G. S., and Scott, E. W. (2012) CCN2/CTGF regulates neovessel formation via targeting structurally conserved cystine knot motifs in multiple angiogenic regulators. FASEB J. 26, 3365–3379 |
| 59 | doi: 10.1016/j.cytogfr.2005.01.004 | Presta, M., Dell’Era, P., Mitola, S., Moroni, E., Ronca, R., and Rusnati, M. (2005) Fibroblast growth factor/fibroblast growth factor receptor system in angiogenesis. *Cytokine Growth Factor Rev.* **16**, 159–178 |
| 60 | doi: 10.1158/1541-7786.MCR-09-0288 | Ptaszynska, M. M., Pendrak, M. L., Stracke, M. L., and Roberts, D. D. (2010) Autotaxin Signaling via Lysophosphatidic Acid Receptors Contributes to Vascular Endothelial Growth Factor–Induced Endothelial Cell Migration. *Mol. Cancer Res.* **8**, 309–321 |
| 61 | doi: 10.1073/pnas.0611653104 | Rangel, R., Sun, Y., Guzman-Rojas, L., Ozawa, M. G., Sun, J., Giordano, R. J., Van Pelt, C. S., Tinkey, P. T., Behringer, R. R., Sidman, R. L., Arap, W., and Pasqualini, R. (2007) Impaired angiogenesis in aminopeptidase N-null mice. Proc. Natl. Acad. Sci. 104, 4588–4593 |
| 62 | doi: 10.18632/oncotarget.17718 | dos Santos, S. N., Sheldon, H., Pereira, J. X., Paluch, C., Bridges, E. M., El-Cheikh, M. C., Harris, A. L., and Bernardes, E. S. (2017) Galectin-3 acts as an angiogenic switch to induce tumor angiogenesis via Jagged-1/Notch activation. Oncotarget. 8, 49484–49501 |
| 63 | doi: 10.3389/fcell.2022.852989 | Subramaniam, S., Liu, J., Fletcher, C., Ramchandran, R., and Weiler, H. (2022) Coagulation Factor IIIa (f3a) Knockdown in Zebrafish Leads to Defective Angiogenesis and Mild Bleeding Phenotype. *Front. Cell Dev. Biol.* **10**, 1–9 |
| 64 | doi: 10.1038/labinvest.3700594 | Sullivan, K. M., Bissonnette, R., Yanagisawa, H., Hussain, S. N., and Davis, E. C. (2007) Fibulin-5 functions as an endogenous angiogenesis inhibitor. *Lab. Investig.* **87**, 818–827 |
| 65 | doi: 10.1074/jbc.M110673200 | Tang, H., Hao, Q., Fitzgerald, T., Sasaki, T., Landon, E. J., and Inagami, T. (2002) Pyk2/CAKβ Tyrosine Kinase Activity-mediated Angiogenesis of Pulmonary Vascular Endothelial Cells. *J. Biol. Chem.* **277**, 5441–5447 |
| 66 | doi: 10.1093/carcin/bgh171 | Tangkeangsirisin, W. (2004) PC cell-derived growth factor (PCDGF/GP88, progranulin) stimulates migration, invasiveness and VEGF expression in breast cancer cells. *Carcinogenesis*. **25**, 1587–1592 |
| 67 | doi: 10.14670/HH-24.1287 | Tanriover, G., Seval, Y., Sati, L., Gunel, M., and Demir, N. (2009) CCM2 and CCM3 proteins contribute to vasculogenesis and angiogenesis in human placenta. *Histol. Histopathol.* **24**, 1287–94 |
| 68 | doi: 10.1074/jbc.M115.708446 | Towner, L. D., Wheat, R. A., Hughes, T. R., and Morgan, B. P. (2016) Complement Membrane Attack and Tumorigenesis. *J. Biol. Chem.* **291**, 14927–14938 |
| 69 | doi: 10.1038/sj.emboj.7601589 | Toyofuku, T., Yabuki, M., Kamei, J., Kamei, M., Makino, N., Kumanogoh, A., and Hori, M. (2007) Semaphorin-4A, an activator for T-cell-mediated immunity, suppresses angiogenesis via Plexin-D1. *EMBO J.* **26**, 1373–1384 |
| 70 | PMID: 21036706 | Uramoto, H., Akyürek, L. M., and Hanagiri, T. (2010) A positive relationship between filamin and VEGF in patients with lung cancer. *Anticancer Res.* **30**, 3939–44 |
| 71 | doi: 10.1002/jcp.30308 | Vega‐Mendoza, D., Cañas‐Linares, A., Flores‐Alcantar, A., Espinosa‐Neira, R., Melchy‐Perez, E., Vera‐Estrella, R., Auvynet, C., and Rosenstein, Y. (2021) CD43 (sialophorin) is involved in the induction of extracellular matrix remodeling and angiogenesis by lung cancer cells. J. Cell. Physiol. 236, 6643–6656 |
| 72 | doi: 10.1080/07357900802392675 | Voigt, H., Vetter-Kauczok, C. S., Schrama, D., Hofmann, U. B., Becker, J. C., and Houben, R. (2009) CD147 Impacts Angiogenesis and Metastasis Formation. *Cancer Invest.* **27**, 329–333 |
| 73 | doi: 10.1038/nature09002. | Wang, Y., Nakayama, M., Pitulescu, M. E., Schmidt, T. S., Bochenek, M. L., Sakakibara, A., Adams, S., Davy, A., Deutsch, U., Lüthi, U., Barberis, A., Benjamin, L. E., Mäkinen, T., Nobes, C. D., and Adams, R. H. (2010) Ephrin-B2 controls VEGF-induced angiogenesis and lymphangiogenesis. Nature. 465, 483–486 |
| 74 | doi.org/10.1016/j.prp.2019.152531 | Wang, H., Chen, H., Jiang, Z., Lin, Y., Wang, X., Xiang, J., and Peng, J. (2019) Integrin subunit alpha V promotes growth, migration, and invasion of gastric cancer cells. *Pathol. - Res. Pract.* **215**, 152531 |
| 75 | doi: 10.1038/sj.onc.1209264 | Wu, M., Wallace, M. R., and Muir, D. (2006) Nf1 haploinsufficiency augments angiogenesis. *Oncogene*. **25**, 2297–2303 |
| 76 | doi: 10.1167/iovs.05-1528. | Xu, Z., Yu, Y., and Duh, E. J. (2006) Vascular Endothelial Growth Factor Upregulates Expression of ADAMTS1 in Endothelial Cells through Protein Kinase C Signaling. *Investig. Opthalmology Vis. Sci.* **47**, 4059 |
| 77 | doi: 10.1093/cvr/cvm085 | Xu, H., Czerwinski, P., Hortmann, M., Sohn, H.-Y., Forstermann, U., and Li, H. (2008) Protein kinase C promotes angiogenic activity of human endothelial cells via induction of vascular endothelial growth factor. *Cardiovasc. Res.* **78**, 349–355 |
| 78 | doi: 10.1073/pnas.0901324106 | Yi, M., and Schnitzer, J. E. (2009) Impaired tumor growth, metastasis, angiogenesis and wound healing in annexin A1-null mice. *Proc. Natl. Acad. Sci.* **106**, 17886–17891 |
| 79 | doi: 10.1038/nm.2928 | Yoshioka, K., Yoshida, K., Cui, H., Wakayama, T., Takuwa, N., Okamoto, Y., Du, W., Qi, X., Asanuma, K., Sugihara, K., Aki, S., Miyazawa, H., Biswas, K., Nagakura, C., Ueno, M., Iseki, S., Schwartz, R. J., Okamoto, H., Sasaki, T., Matsui, O., Asano, M., Adams, R. H., Takakura, N., and Takuwa, Y. (2012) Endothelial PI3K-C2α, a class II PI3K, has an essential role in angiogenesis and vascular barrier function. Nat. Med. 18, 1560–1569 |
| 80 | doi: 10.1096/fj.08-113860 | Zhang, J., Cao, R., Zhang, Y., Jia, T., Cao, Y., and Wahlberg, E. (2009) Differential roles of PDGFR‐α and PDGFR‐β in angiogenesis and vessel stability. *FASEB J.* **23**, 153–163 |
| 81 | doi.org/10.1186/s12935-019-1027-3 | Zhang, R., Liu, Q., Li, T., Liao, Q., and Zhao, Y. (2019) Role of the complement system in the tumor microenvironment. *Cancer Cell Int.* **19**, 300 |
| 82 | doi: 10.1177/08968608221116956 | Zhang, P., Miyata, K. N., Nast, C. C., LaPage, J. A., Mahoney, M., Nguyen, S., Khan, K., Wu, Q., Adler, S. G., and Dai, T. (2023) Dual therapy with an angiotensin receptor blocker and a JAK1/2 inhibitor attenuates dialysate-induced angiogenesis and preserves peritoneal membrane structure and function in an experimental CKD rat model. Perit. Dial. Int. J. Int. Soc. Perit. Dial. 43, 159–167 |
| 83 | doi: 10.1161/CIRCRESAHA.109.195156 | Zheng, Y., Vertuani, S., Nyström, S., Audebert, S., Meijer, I., Tegnebratt, T., Borg, J.-P., Uhlén, P., Majumdar, A., and Holmgren, L. (2009) Angiomotin-Like Protein 1 Controls Endothelial Polarity and Junction Stability During Sprouting Angiogenesis. Circ. Res. 105, 260–270 |
| 84 | doi: 10.1161/ATVBAHA.113.303053 | Zhou, H. J., Chen, X., Huang, Q., Liu, R., Zhang, H., Wang, Y., Jin, Y., Liang, X., Lu, L., Xu, Z., and Min, W. (2014) AIP1 Mediates Vascular Endothelial Cell Growth Factor Receptor-3–Dependent Angiogenic and Lymphangiogenic Responses. Arterioscler. Thromb. Vasc. Biol. 34, 603–615 |
| 85 | doi: 10.3389/fonc.2014.00087 | Wallace, J. A., Pitarresi, J. R., Sharma, N., Palettas, M., CuitiÃ±o, M. C., Sizemore, S. T., Yu, L., Sanderlin, A., Rosol, T. J., Mehta, K. D., Sizemore, G. M., and Ostrowski, M. C. (2014) Protein Kinase C Beta in the Tumor Microenvironment Promotes Mammary Tumorigenesis. Front. Oncol. 4, 1–10 |
| 86 | doi: 10.1007/s10456-016-9498-5 | Koo, Y., Barry, D. M., Xu, K., Tanigaki, K., Davis, G. E., Mineo, C., and Cleaver, O. (2016) Rasip1 is essential to blood vessel stability and angiogenic blood vessel growth. *Angiogenesis*. **19**, 173–190 |
| 87 | doi: 10.7150/ijbs.65653 | Wang, M., Zhang, C., Zheng, Q., Ma, Z., Qi, M., Di, G., Ling, S., Xu, H., Qi, B., Yao, C., Xia, H., and Jiang, X. (2022) RhoJ facilitates angiogenesis in glioblastoma via JNK/VEGFR2 mediated activation of PAK and ERK signaling pathways. Int. J. Biol. Sci. 18, 942–955 |
| 88 | doi: 10.1038/ncb3376 | Banh, R. S., Iorio, C., Marcotte, R., Xu, Y., Cojocari, D., Rahman, A. A., Pawling, J., Zhang, W., Sinha, A., Rose, C. M., Isasa, M., Zhang, S., Wu, R., Virtanen, C., Hitomi, T., Habu, T., Sidhu, S. S., Koizumi, A., Wilkins, S. E., Kislinger, T., Gygi, S. P., Schofield, C. J., Dennis, J. W., Wouters, B. G., and Neel, B. G. (2016) PTP1B controls non-mitochondrial oxygen consumption by regulating RNF213 to promote tumour survival during hypoxia. Nat. Cell Biol. 18, 803–813 |
| 89 | doi: 10.1016/j.celrep.2019.04.034 | Demian, W. L., Persaud, A., Jiang, C., Coyaud, É., Liu, S., Kapus, A., Kafri, R., Raught, B., and Rotin, D. (2019) The Ion Transporter NKCC1 Links Cell Volume to Cell Mass Regulation by Suppressing mTORC1. *Cell Rep.* **27**, 1886-1896.e6 |
| 90 | doi: 10.1038/nature12345 | Wang, X., Abraham, S., McKenzie, J. A. G., Jeffs, N., Swire, M., Tripathi, V. B., Luhmann, U. F. O., Lange, C. A. K., Zhai, Z., Arthur, H. M., Bainbridge, J. W. B., Moss, S. E., and Greenwood, J. (2013) LRG1 promotes angiogenesis by modulating endothelial TGF-β signalling. Nature. 499, 306–311 |
| 91 | doi: 10.1093/abbs/gmv131 | Sheng, J., and Xu, Z. (2016) Three decades of research on angiogenin: a review and perspective. *Acta Biochim. Biophys. Sin. (Shanghai).* **48**, 399–410 |
